# Toward sustainable energy production: a comparative machine learning framework for predicting green hydrogen cost across the african continent

**DOI:** 10.1038/s41598-026-47726-w

**Published:** 2026-04-17

**Authors:** Ashraf M. T. Elewa, Moustafa Gamal Snousy, Ahmed M. Saqr, Hussein M. Elshafie, Ashraf R. Abouelmagd, Ali Mahmoud Hussain, Tarek Abd El-Hafeez

**Affiliations:** 1 Geology Department, Faculty of Science, Minia University, El-Minia, 61519 Egypt; 2 Egyptian Petroleum Sector, Petrotrade Co., Block 10 - Plot No. 3 - District 11, Nasr City, Cairo Egypt; 3 Irrigation and Hydraulics Department, Faculty of Engineering, Mansoura University, Mansoura, 35516 Egypt; 4 Department of Computer Science, Faculty of Computers and Information, Luxor University, Luxor, 85951 Egypt; 5 Egyptian Petroleum Sector, Egyptian Natural Gas Holding Company, 85 Nasr Road, 1 st District, Nasr City, Cairo Egypt; 6 Department of Computer Science, Faculty of Science, EL- Minia, Minia University, Minia, Egypt

**Keywords:** Green hydrogen, Levelized Cost of Hydrogen (LCOH), Artificial intelligence, Gradient boosting, SHAP analysis, Sustainable Development Goals (SDGs), Energy science and technology, Engineering, Environmental sciences, Environmental social sciences, Mathematics and computing

## Abstract

Rapid decarbonisation of hard-to-electrify sectors requires low-emission hydrogen, but deployment is constrained by uncertainty in the Levelized Cost of Hydrogen (LCOH) across diverse national contexts. Using Africa as a case study, where green hydrogen planning spans highly heterogeneous conditions, this study develops a comparative machine learning framework for country-scale cost screening before major infrastructure commitments. A harmonized dataset of 54 African scenarios was compiled, with LCOH (EUR/kg) as the target variable and 14 predictors capturing project scale, renewable capacity, storage and transport infrastructure, investment and maturity stage, energy security and sustainability indices, market variables, CO_2_ reduction potential, and water demand. The workflow integrated exploratory statistics, preprocessing, and systematic benchmarking of 11 regression models using an independent 20% holdout split, complemented by repeated nested cross-validation. Across the compiled cases, LCOH ranged from 3.75 to 5.60 EUR/kg with a median of 4.90 EUR/kg, showing clear cost stratification by project maturity stage. Holdout validation identified Hyperopt optimized Gradient Boosting as the best performing model (R^2^ = 0.9762, RMSE = 0.0840 EUR/kg, MAE = 0.0663 EUR/kg), followed closely by Bayesian tuned XGBoost (R^2^ = 0.9713). Nested cross-validation confirmed model stability (Hyperopt_GB: R^2^ = 0.9710 ± 0.032). SHAP analysis revealed that renewable energy capacity, electrolyser capacity, and the energy security index contributed most to predicted LCOH variability within the dataset. The framework provides a transferable screening pipeline for prioritizing investment and data collection in data-scarce settings, with explicit linkages to Sustainable Development Goal (SDG) relevant indicators, including energy security, climate mitigation, and water stress. This approach complements deterministic techno-economic appraisal by enabling rapid cross-country comparison during early-stage planning Fig.S1.

**Supplementary Information:**

The online version contains supplementary material available at 10.1038/s41598-026-47726-w.

## Introduction

Hydrogen is being reconsidered as an option for reducing carbon emissions, given the difficulty of rapidly and widely electrifying many high-temperature, long-distance applications^1^. However, the current hydrogen economy remains dominated by conventional demand and supply channels^2^. The International Energy Agency notes that global hydrogen use is still concentrated in traditional sectors such as refining and chemicals, and is largely met by hydrogen produced from unprocessed fossil fuels^3^. This reality dictates a clear deployment sequence. First, low-emission hydrogen must replace high-emission hydrogen in existing demand centers. Then, it must expand to new end-use applications where hydrogen-based fuels or feedstocks can achieve emission reductions that are difficult to achieve otherwise^4^. These shifts underscore the importance of early cost assessment, as infrastructure decisions are difficult to reverse once capital has been allocated^5^.

Africa is often portrayed as a region brimming with opportunities, given the convergence of its renewable resource potential with pressing development priorities^6^. However, translating resource maps into bankable hydrogen projects hinges on these projects’ ability to achieve a competitive Levelized Cost of Hydrogen (LCOH) under local constraints^7^. In this context, LCOH refers to the average cost of hydrogen production over the project’s lifetime, expressed in kilograms, after accounting for the major cost components during the project’s operational period and dividing by total hydrogen production^8^. In the African context, LCOH is rarely determined by a single factor but rather shaped by a complex interplay of factors related to project scale and system readiness^9^. The dataset compiled for this study reflects these interactions by linking LCOH to indicators such as electrolyzer capacity, hydrogen production capacity, renewable energy capacity, storage capacity, pipeline length, investment, and project maturity stage^10^. It also incorporates enabling and inhibiting variables that are often crucial in assessing project viability, including energy security, sustainability, export potential, domestic demand, and water demand^11^. These variables vary considerably among African countries. Therefore, continent-wide conclusions require approaches that reflect country-level variation rather than assuming a single typical scenario^12^.

Many published studies on hydrogen costing rely on techno-economic analysis and deterministic scenario assumptions, which remain essential for detailed project design^13^. This limitation emerges early in the decision-making process, when planners need to rank multiple countries or concepts using incomplete information^14^. At this stage, cost factors may interact non-linearly, and uncertainty is high across multiple dimensions simultaneously. Additionally, it was evident from the literature that a data-driven approach can complement traditional assessment by directly extracting multivariate relationships from a structured set of indicators^15^. However, a methodological gap remains in hydrogen cost studies in Africa^16,17^. Cross-country studies often apply a single model without demonstrating its robustness to small- to medium-sized tabular datasets. Another gap is interpretability. Decision-makers need cost drivers that can be communicated, discussed, and updated as projects evolve^18^. A third gap concerns alignment with the Sustainable Development Goals (SDGs)^1^, as cost projections are often reported without explicitly linking them to the sustainability dimensions already represented in available indicators, such as energy security, sustainability assessment, Carbon Dioxide (CO_2_) reduction potential, and water demand^19^.

A country-scale comparative Machine Learning (ML) framework is developed to screen green-hydrogen cost across Africa using a standardized indicator table. The analysis is conducted on 54 African scenarios. In each scenario, LCOH is used as the target variable, and a consistent set of predictors is used to represent project scale, renewable supply, storage and transport infrastructure, investment and maturity stage, market orientation, enabling context, and resource constraints. Recent techno-economic and geospatial assessments have provided detailed LCOH estimates^20^, and recent ML studies have explored hydrogen-related prediction tasks^21^. The present study is not intended to replace techno-economic appraisal or geospatial optimization. Instead, a standardized small-tabular-data benchmarking framework is provided for country-scale screening under data scarcity, supported by robust evaluation and interpretable outputs. First, a continent-scale, harmonized scenario-table structure is provided so that consistent cross-country comparison can be performed within a single modeling pipeline. Second, multiple regression models are benchmarked under a unified preprocessing and evaluation protocol to support model selection under small-sample conditions. Third, interpretability is integrated so that predictive patterns are translated into decision-relevant screening signals rather than being presented as purely computational outputs. Finally, SDG alignment is treated as a structured evidence map based on SDG-target coverage, and selected targets are linked to the same indicators used for prediction and interpretation. In this way, a transparent bridge is provided between cost screening and development-oriented criteria used by policy and finance stakeholders.

## Materials and methods

**Fig. 1:**
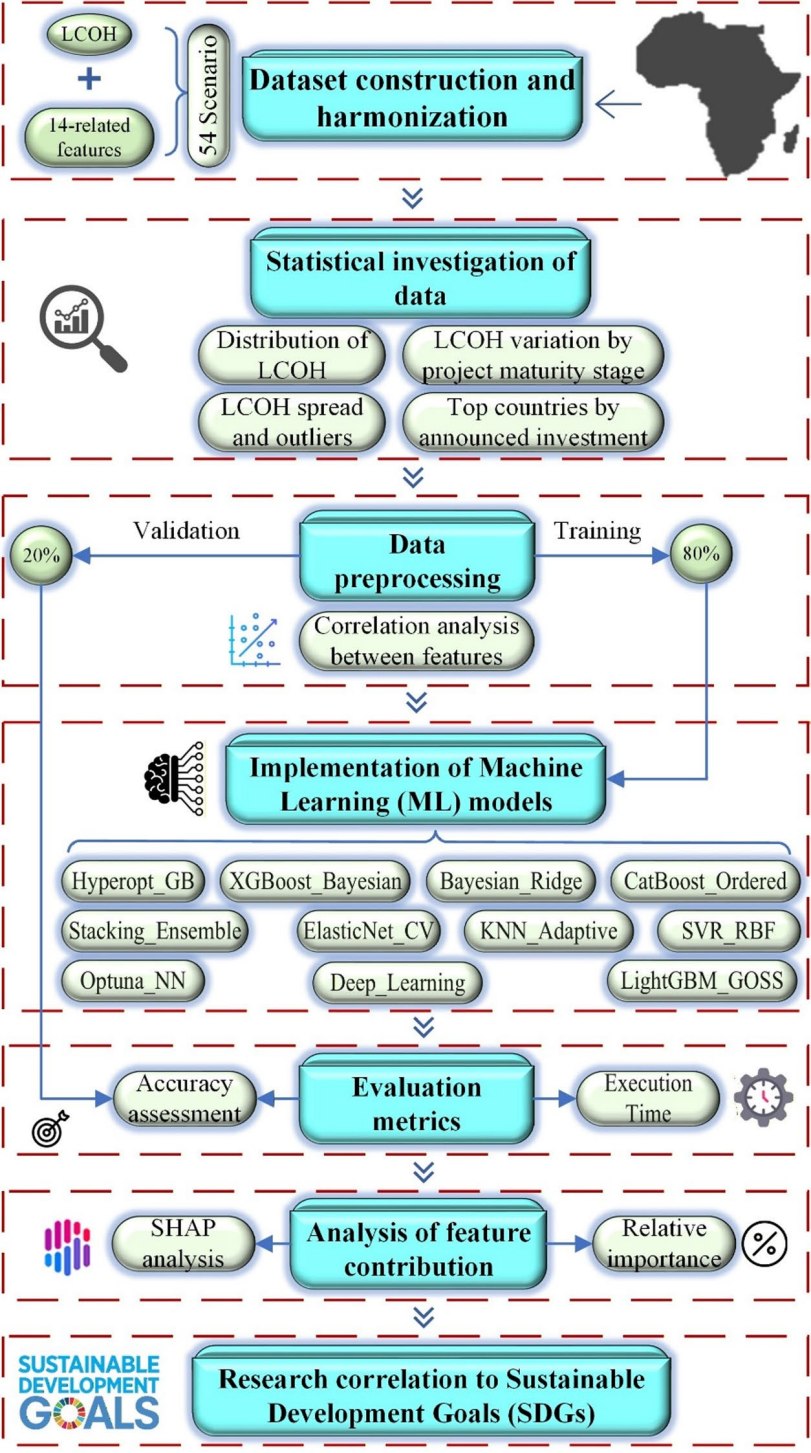
A flowchart of the research methodology.

Figure 1 summarizes the overall systematic workflow as a step-by-step sequence linking data collection, model development, validation, interpretation, and sustainability mapping into a single, reproducible procedure. The “Materials and Methods” section first describes the collection of a country-wide dataset for 54 African scenarios, where the LCOH (EUR/kg) is defined as the target variable, and the predictive variables represent project size, infrastructure readiness, enabling context, market orientation, maturity stage, and water demand. The workflow then outlines the statistical analysis used to characterize the target distribution, validate variable ranges, and document the maturity-related structure prior to modeling. Finally, the preprocessing steps for converting the collected table into an ML-ready matrix are described, including unit consistency verification, categorical descriptor encoding, and feature scaling where required. The subsequent subsections present the application of multiple ML regression models to predict LCOH within a consistent modular design, followed by the evaluation metrics and validation logic used to ensure a fair comparison. Finally, the methodology presents a SHapley Additive exPlanations (SHAP) to interpret feature contributions and an SDG correlation step that links evidence from the modeling workflow to SDG themes using a traceable SDG evidence matrix.

### Dataset construction and harmonization

The dataset was designed to support country-level learning in green hydrogen economics using a standardized attribute scheme (Table 1). Each record was defined as one scenario. 54 scenarios across African countries were included for statistical and ML analyses. Alternative assumptions were retained as separate observations when scenario conditions materially changed (for example, “Tanzania” and “Tanzania_alt”). The learning task was kept at the scenario level. A single value per country was not used.

The country-level dataset was a harmonized indicator-based scenario table. It was not raw historical data. Values were compiled from national hydrogen strategies, international assessments, and project-level reports. Values included LCOH, electrolyzer capacity, hydrogen production, renewable energy capacity, CO₂ reduction, storage capacity, distribution pipeline length, energy security score, sustainability index, export potential, domestic demand, investment, project maturity stage, and water demand. All values were converted to common units (GW, Mtpa, EUR/kg, Mt/year, Mm³, km, USD billion). Values were mapped to unified variables in Table 1. Harmonization used three steps. Multiple source values used the priority rule: (i) official national strategies, (ii) international assessments, (iii) project reports. Discrepancies within ± 20% were averaged. Missing values used region-specific ratios (electrolyzer/renewable GW, H₂/GW electrolysis, water/Mt H₂) from better-documented regional peers. Numerical indicators were z-score standardized pre-training. Values were denormalized for reporting. “Tanzania/Tanzania_alt” were separate rows for alternative futures. Transformations were: (i) direct sources, (ii) linear interpolation/extrapolation, (iii) regional ratios for similar countries. The full dataset is introduced in Supplementary Table S1. Additionally, Python code for compilation, harmonization, and generation is illustrated in the supplementary material. These files reproduce all analyses.

For each scenario, LCOH (EUR/kg) was the target variable. Fourteen techno-economic variables described project scale, infrastructure, enabling context, market orientation, and resource constraints. Variables were electrolyzer capacity (GW), renewable energy capacity (GW), storage capacity (Mm³), distribution pipeline length (km), investment (billion USD), project maturity stage, export potential (Mtpa), domestic demand (Mtpa), hydrogen production capacity (Mtpa), CO₂ reduction (Mt/year), water demand (Mm³/year), energy security score, and sustainability index. Variable definitions, units, and transformations are in Table S1. Table S1 shows variable-by-variable lineage.

Several variables were scenario-constructed. Base and derived variables were distinguished to avoid overstated performance. Base variables were directly sourced. Derived variables were computed. Hydrogen production capacity was from electrolyzer capacity at 70% load factor. CO₂ reduction was from hydrogen output using a 9.3 tCO₂/tH₂ factor. Export potential, domestic demand, and water demand were % of hydrogen output. Derivations are declared in Table S1. Python code implements them transparently.

Two composite indicators were included. The Energy Security Score reflected energy system diversification and robustness. It had four sub-components: (i) primary energy diversity, (ii) domestic renewable share, (iii) import dependency, and (iv) grid reliability. Each sub-indicator used min-max normalization (0–10 scale). The overall score was equal-weight mean. The Sustainability Index aggregated sustainability dimensions. Inputs were mapped “higher-is-better”. They were min-max scaled (0–10). Final index was equal-weight geometric mean. This reduced full compensation. Full definitions are in Table S1 and the Python script. Project maturity stage used ordinal encoding: Concept = 1, Feasibility = 2, FEED/FID = 3, FID/Construction = 4, Multiple Stages = 5.

Finally, robustness against deterministic feature relationships was assessed by rerunning the clustering workflow using (i) the full predictor set and (ii) a base-only set in which derived variables were excluded. The resulting PCA + K-means (k = 3) structures were found to remain qualitatively consistent; the comparison was provided in Figs. S1–S2 (full vs. base-only).

**Table 1:** Compiled African dataset reporting Levelized Cost of Hydrogen (LCOH), and the main influencing parameters used in this study^22–24^.

Country	LCOH (EUR/kg)	Electrolyzer Capacity (GW)	H_2_ Production Capacity (Mtpa)	Renewable Energy Capacity (GW)	CO_2_ Reduction (Mt per year)	Storage Capacity (Mm^3^)	Distribution Pipeline (km)	Energy Security Score	Sustainability Index	Export Potential (Mtpa)	Domestic Demand (Mtpa)	Investment (Billion USD)	Project Maturity Stage	Water Demand (Mm^3^ per year)
Algeria	4.0	3.5	0.7	12.0	15.0	10.0	700.0	7.3	7.8	0.4	0.3	3.5	Concept	35.0
Angola	4.9	0.3	0.08	2.0	1.5	2.0	150.0	6.2	7.5	0.05	0.03	1.5	Feasibility	5.0
Benin	4.9	0.4	0.08	1.5	1.5	2.0	220.0	6.5	8.0	0.06	0.02	1.0	Feasibility	4.0
Botswana	4.3	1.5	0.3	4.5	5.0	6.0	350.0	7.0	8.2	0.2	0.1	2.0	Feasibility	15.0
Burkina Faso	5.1	0.3	0.08	1.2	1.2	1.5	150.0	5.9	7.2	0.03	0.05	0.5	Concept	4.0
Burundi	5.2	0.12	0.025	0.45	0.45	0.8	100.0	5.5	7.2	0.015	0.02	0.3	Concept	1.5
Cameroon	4.8	0.4	0.1	1.5	1.8	2.0	200.0	6.2	7.5	0.05	0.05	0.9	Concept	5.0
Cape Verde	5.4	0.1	0.02	0.3	0.3	0.5	60.0	5.0	6.8	0.01	0.01	0.2	Concept	1.0
Central African Republic	5.4	0.18	0.035	0.6	0.6	1.0	140.0	5.8	7.5	0.02	0.015	0.4	Concept	2.0
Chad	5.5	0.2	0.04	0.8	0.8	1.2	160.0	6.0	7.8	0.03	0.02	0.5	Concept	2.5
Comoros	5.2	0.15	0.03	0.4	0.4	0.6	80.0	5.8	7.2	0.02	0.01	0.3	Concept	1.5
Congo	4.9	0.3	0.08	1.0	1.2	1.5	150.0	6.0	7.2	0.04	0.04	0.6	Concept	4.0
Democratic Republic of Congo	5.1	0.2	0.05	0.6	0.8	1.0	100.0	5.5	6.8	0.02	0.03	0.4	Concept	2.5
Djibouti	4.2	2.0	0.4	5.0	7.0	8.0	450.0	7.8	8.5	0.3	0.1	3.5	Feasibility	20.0
Egypt	4.2	1.2	0.3	8.2	8.5	12.0	600.0	7.5	8.0	0.15	0.15	5.0	Multiple Stages	25.0
Eritrea	5.0	0.3	0.08	1.0	1.0	1.5	150.0	6.0	7.5	0.05	0.03	0.8	Concept	4.0
Eswatini	5.1	0.2	0.04	0.6	0.6	0.8	100.0	5.9	7.2	0.02	0.02	0.4	Concept	2.0
Ethiopia	4.6	0.5	0.12	3.5	2.0	3.0	200.0	6.8	8.5	0.02	0.1	0.8	Concept	8.0
Gabon	4.5	0.6	0.15	2.0	2.5	3.0	250.0	6.8	8.0	0.1	0.05	1.2	Concept	7.5
Gambia	5.3	0.15	0.03	0.5	0.5	0.8	90.0	5.5	7.0	0.02	0.01	0.3	Concept	1.5
Ghana	4.6	1.0	0.2	3.0	3.5	4.0	350.0	6.8	8.0	0.12	0.08	1.8	Feasibility	10.0
Guinea	5.1	0.35	0.07	1.2	1.2	1.5	180.0	6.2	7.8	0.04	0.03	0.8	Feasibility	3.5
Guinea Bissau	5.5	0.1	0.02	0.3	0.3	0.5	60.0	5.2	6.8	0.01	0.01	0.2	Concept	1.0
Ivory Coast	4.7	0.8	0.18	2.5	3.0	3.5	300.0	6.5	7.8	0.1	0.08	1.5	Feasibility	9.0
Kenya	4.8	0.8	0.2	5.0	3.5	5.0	300.0	6.5	8.8	0.05	0.15	1.2	Feasibility	10.0
Lesotho	5.0	0.25	0.05	0.8	0.8	1.0	120.0	6.2	7.5	0.03	0.02	0.5	Concept	2.5
Liberia	5.3	0.25	0.05	0.8	0.8	1.0	150.0	6.0	7.5	0.03	0.02	0.5	Concept	2.5
Libya	4.7	0.6	0.15	2.0	2.5	3.0	180.0	6.5	7.8	0.08	0.07	1.0	Concept	8.0
Madagascar	4.9	0.5	0.12	2.0	2.0	2.5	250.0	6.8	8.2	0.08	0.04	1.0	Feasibility	6.0
Malawi	5.0	0.3	0.06	0.8	1.0	1.2	140.0	5.8	7.0	0.03	0.03	0.6	Concept	3.0
Mali	5.2	0.5	0.12	2.0	2.0	2.5	200.0	6.2	7.5	0.06	0.06	0.8	Concept	7.0
Mauritania	3.8	15.0	2.5	30.0	45.0	28.0	850.0	8.5	9.0	2.2	0.3	40.0	FEED/FID	180.0
Mauritius	4.8	0.4	0.1	1.5	1.5	2.0	200.0	6.5	8.0	0.06	0.04	0.8	Feasibility	5.0
Morocco	4.1	2.5	0.6	14.0	12.0	18.0	950.0	8.0	8.5	0.5	0.1	8.0	Feasibility	45.0
Mozambique	4.6	0.7	0.14	2.2	2.5	3.0	320.0	6.5	7.8	0.08	0.06	1.4	Feasibility	7.0
Namibia	4.1	5.5	1.2	11.0	22.0	12.0	500.0	8.2	9.2	1.0	0.2	9.4	FID/Construction	60.0
Niger	5.3	0.2	0.05	0.8	0.8	1.0	100.0	5.5	7.0	0.02	0.03	0.3	Concept	3.0
Nigeria	4.4	2.0	0.4	6.0	8.0	8.0	600.0	7.2	8.2	0.3	0.1	4.0	Feasibility	20.0
Rwanda	5.2	0.25	0.05	1.2	1.5	2.0	200.0	6.5	7.8	0.02	0.03	0.9	Concept	4.5
Sao Tome	5.6	0.08	0.02	0.25	0.25	0.4	50.0	5.0	6.5	0.01	0.01	0.15	Concept	0.8
Senegal	4.5	0.7	0.15	3.0	3.0	3.5	280.0	6.8	8.0	0.08	0.07	1.4	Feasibility	10.0
Seychelles	5.5	0.1	0.02	0.3	0.3	0.5	50.0	5.2	6.8	0.01	0.01	0.2	Concept	1.0
Sierra Leone	5.2	0.2	0.04	0.6	0.6	0.8	120.0	5.8	7.2	0.02	0.02	0.4	Concept	2.0
Somalia	5.3	0.2	0.05	0.6	0.6	1.0	100.0	5.5	7.0	0.02	0.03	0.4	Concept	2.5
South Africa	4.5	2.0	0.4	6.5	7.0	8.0	400.0	7.8	8.0	0.2	0.2	4.6	Feasibility	20.0
South Sudan	5.4	0.15	0.03	0.4	0.5	0.6	80.0	5.0	6.8	0.01	0.02	0.2	Concept	1.5
Sudan	5.0	0.4	0.1	1.5	1.5	2.0	120.0	5.8	7.2	0.04	0.06	0.6	Concept	5.0
*Tanzania	4.7	0.9	0.18	3.0	3.5	4.0	400.0	6.8	8.0	0.12	0.06	1.8	Feasibility	9.0
*Tanzania alt	4.5	0.7	0.14	2.5	2.5	3.0	320.0	6.8	8.0	0.08	0.08	1.6	Feasibility	7.0
Togo	5.0	0.3	0.06	1.0	1.0	1.5	160.0	6.0	7.5	0.04	0.02	0.7	Concept	3.0
Tunisia	4.4	1.0	0.25	4.0	4.0	4.0	250.0	7.0	8.0	0.1	0.15	2.0	Concept	15.0
Uganda	5.1	0.35	0.07	1.5	2.0	2.5	250.0	6.2	7.5	0.04	0.03	1.2	Feasibility	5.5
Zambia	4.8	0.5	0.1	1.8	1.8	2.0	250.0	6.2	7.5	0.06	0.04	1.0	Concept	5.0
Zimbabwe	4.9	0.4	0.08	1.0	1.2	1.5	180.0	6.0	7.2	0.04	0.04	0.8	Concept	4.0

*Note: “alt” denotes an alternative scenario for the same country (Tanzania) under different assumptions (e.g., lower vs. higher investment). Both scenarios are intentionally kept as separate observations because all ML and statistical analyses were performed using the disaggregated records.

### Statistical investigation of data

Statistical analysis of the data was conducted as a systematic exploratory step to characterize the target variable, verify data quality, and determine the clustering schemes to be used later in the modeling^25^. The LCOH values ​​(EUR/kg) underwent initial screening using univariate distribution diagnostic tools. A histogram with an added density curve was used to examine the central tendency, dispersion, and potential skewness of the target variable. A box plot was then constructed to summarize the median, interquartile range, and outliers, and to identify observations that might be considered anomalies under robust measurement and loss functions. To examine whether the cost variance corresponded to development readiness, the dataset was segmented according to the recorded project maturity stage for each scenario. A clustered box plot was created across the maturity categories shown in the dataset, which include concept stage, feasibility study, multi-stage, final investment decision/construction stage, and preliminary engineering design/final investment decision stage. This step was used to verify whether maturity imparted a systematic structure to the target distribution and to confirm the suitability of the maturity field for subsequent coding and interpretation. In parallel, reported investment (in billions of US dollars) was used to construct a country ranking. The study filtered the dataset to include the top ten investments and presented them graphically using a horizontal bar chart. This process supported a simple concentration check within the investment field and ensured that outliers were transparently represented before preprocessing decisions were made.

### Data preprocessing

Preprocessing transformed the aggregated African scenario dataset into an ML-ready matrix while preserving the objective definition^26^. The aggregated table contains a single objective column (LCOH, in EUR/kg) and fourteen predictors describing project size, infrastructure, enabling context, and resource demand. The records were first validated for structural consistency, including unit coherence across numeric fields (e.g., gigawatts, million tons per year, kilometers, billion US dollars, and million cubic meters per year) and the presence of taxonomic entries for project maturity stage. The taxonomic attributes were then numerically converted to ensure compatibility with the full suite of ML algorithms. The country was treated as a categorical context variable. Its numeric representation as an ordinal or distance-based quantity was not interpreted. To avoid artificial ordering effects and to reduce leakage risk, we used regularized target encoding computed within cross-validation folds. This approach assigns a smoothed country-level target statistic based only on training folds, and it prevents direct exposure of validation targets during encoding. The purpose of this variable is to capture residual context that may reflect unobserved country-level factors (e.g., institutional conditions, policy context, geography) that are not explicitly represented by the techno-economic predictors. After coding, the dataset was divided into independent subsets for model development and final evaluation, with an 80% allocation for training and 20% for testing. This division was applied before any model adjustment transformations to avoid interference from the test set data and to ensure a fair evaluation of the unseen data. For feature-size-sensitive algorithms (such as distance-based, kernel-based, and neural models), numerical predictors were scaled using only the parameters extracted from the training set, and the same transformation was then applied to the test set without adjustment. The resulting training matrix (features) and objective vector were used to fine-tune the model and calibrate hyperparameters, while the test set was retained until the final performance report to ensure that the evaluation reflected generalization rather than retention. As a preprocessing-linked diagnostic step, pairwise linear associations among LCOH and the study predictors were computed using Pearson’s correlation coefficient and summarized as a correlation heatmap. Pearson’s r was computed as (Eq. 1)^27^:

1 $$\:\mathrm{r}=\frac{\sum\:_{\mathrm{i}=1}^{\mathrm{n}}(\mathrm{c}-{\widehat{\mathrm{c}}}_{\mathrm{i}})({\mathrm{d}}_{\mathrm{i}}-{\widehat{\mathrm{d}}}_{\mathrm{i}})}{\sqrt{\sum\:_{\mathrm{i}=1}^{\mathrm{n}}{({\mathrm{c}}_{\mathrm{i}}-{\widehat{\mathrm{c}}}_{\mathrm{i}})}^{2}}\sqrt{\sum\:_{\mathrm{i}=1}^{\mathrm{n}}{({\mathrm{d}}_{\mathrm{i}}-{\widehat{\mathrm{d}}}_{\mathrm{i}})}^{2}}}$$

where, *c* and *d* denote two variables (e.g., LCOH and one predictor), *n* is the number of scenarios, and $\:{\widehat{c}}_{i}$ and $\:{\widehat{d}}_{i}$ are sample means.

### Implementation of Machine Learning (ML) models

LCOH (EUR/kg) was modeled as a supervised regression target using the compiled predictors describing each African green-hydrogen scenario. The benchmark covered tree-boosting learners, linear regularized regression, instance- and kernel-based regression, neural networks, and a stacking ensemble. All the selected ML techniques were applied to predict LCOH based on the provided 14 input datasets as follows.

#### Hyperopt-optimized Gradient Boosting (Hyperopt_GB)

Hyperopt_GB was implemented as an additive ensemble of regression trees trained sequentially, where each new tree refines the current estimate, as expressed in Eq. (2)^28^.

2 $$\:\widehat{\mathrm{y}}\left(\mathrm{x}\right)={\widehat{\mathrm{y}}}_{0}+{\upeta\:}\sum\:_{\mathrm{m}=1}^{\mathrm{M}}\:{\mathrm{f}}_{\mathrm{m}}\left(\mathrm{x}\right)$$

where, $\:\widehat{y}\left(x\right)$ is the predicted LCOH for scenario *x*, $\:{\widehat{y}}_{0}\:$is the initial constant prediction, *M* is the number of trees, *η* is the learning rate, and fm(⋅) is the *m*-th regression tree.

#### Bayesian-tuned Extreme Gradient Boosting (XGBoost_Bayesian)

XGBoost_Bayesian was implemented as a boosted-tree regressor with hyperparameters tuned using a Bayesian search strategy, while the final model remains an additive ensemble as written in Eq. (3)^29^.

3 $$\:\widehat{\mathrm{y}}\left(\mathrm{x}\right)={\widehat{\mathrm{y}}}_{0}+{\upeta\:}\sum\:_{\mathrm{m}=1}^{\mathrm{M}}\:{f}_{m}\left(x\right)+\sum\:_{\mathrm{m}=1}^{\mathrm{M}}\:{\Omega\:}\left({f}_{m}\right)\:$$

where, $\:{\Omega\:}\left({f}_{m}\right)$ is the tree-regularization term that penalizes model complexity in XGBoost.

#### Bayesian Ridge (Bayesian_Ridge)

Bayesian_Ridge was implemented as a linear probabilistic regressor to provide a stable baseline under coefficient shrinkage, and its prediction is written in Eq. (5)^30^. 

4 $$\:\widehat{\mathrm{y}}\left(\mathrm{x}\right)={\mathrm{x}}^{T}{{\upbeta\:}}_{\mathrm{B}\mathrm{R}}\: \& \:{{\upbeta\:}}_{\mathrm{B}\mathrm{R}}=\mathrm{arg}\underset{{\upbeta\:}}{\mathrm{min}}\left(\parallel\:\mathrm{y}-\mathrm{X}{\upbeta\:}{\parallel\:}_{2}^{2}+{\uplambda\:}\parallel\:{{\upbeta\:}\parallel\:}_{2}^{2}\right)$$

where, *x* and *y* are the training predictors and observed LCOH, $\:{\beta\:}_{BR}$ is the Bayesian-ridge coefficient vector, and *λ* controls *L2* shrinkage.

#### CatBoost Ordered Boosting (CatBoost_Ordered)

CatBoost_Ordered was implemented as a boosting method based on ordered boosting updates to reduce training bias, and the resulting additive predictor is expressed in Eq. (5)^31^.

5 $$\:\widehat{\mathrm{y}}\left(\mathrm{x}\right)={\widehat{\mathrm{y}}}_{0}+{\upeta\:}\sum\:_{\mathrm{t}\:=1}^{\mathrm{T}}\:{\mathrm{f}}_{\mathrm{t}}^{\left({\uppi\:}\right)}\left(\mathrm{x}\right)$$

where, *T* is the number of boosting iterations, $\:{f}_{t}^{\left(\pi\:\right)}$ is the tree built under an ordered (permutation-based) training scheme π.

#### Stacking Ensemble (Stacking_Ensemble)

Stacking_Ensemble was implemented using a two-level structure, where base learners first generate predictions, and a meta-learner combines them into a single estimate, as shown in Eq. (6)^32^.

6 $$\:\widehat{\mathrm{y}}\left(\mathrm{x}\right)=\mathrm{g}({\widehat{\mathrm{y}}}_{1}\left(\mathrm{x}\right),\:{\widehat{\mathrm{y}}}_{2}\left(\mathrm{x}\right),\dots\:,{\widehat{\mathrm{y}}}_{k}\left(\mathrm{x}\right)\:)$$

where, $\:{\widehat{y}}_{k}\left(x\right)$ is the prediction from base learner *k*, and g(⋅) is the meta-learner.

#### Cross-Validated Elastic Net (ElasticNet_CV)

ElasticNet_CV was implemented as a regularized linear regressor, where cross-validation was used to tune the penalty configuration, and the final prediction is written in Eq. (9)^33^.

7 $$\:\widehat{\mathrm{y}}\left(\mathrm{x}\right)={\mathrm{x}}^{T}{{\upbeta\:}}_{\mathrm{E}\mathrm{N}}\:\&\:{{\upbeta\:}}_{\mathrm{E}\mathrm{N}}=\mathrm{arg}\underset{{\upbeta\:}}{\mathrm{min}}\left(\parallel\:\mathrm{y}-\mathrm{X}{\upbeta\:}{\parallel\:}_{2}^{2}+{\upalpha\:}\parallel\:{\upbeta\:}{\parallel\:}_{1}+{\uplambda\:}\parallel\:{{\upbeta\:}\parallel\:}_{2}^{2}\right)$$

where, $\:{\beta\:}_{EN}$ is the elastic-net coefficient vector, and *α* and *λ* control the L1 and L2 penalties selected through cross-validation.

#### Adaptive k-Nearest Neighbors (KNN_Adaptive)

KNN_Adaptive was implemented as a local regression method that estimates LCOH from the responses of the most similar scenarios in the feature space, as expressed in Eq. (8)^32^.

8 $$\:\widehat{\mathrm{y}}\left(\mathrm{x}\right)=\frac{1}{k}\sum\:_{i\:\in\:\:Nk\left(x\right)}{y}_{i}$$

where, *k* is the neighborhood size, *Nk(x)* is the index set of the *k* nearest training samples to *x*, and $\:{y}_{i}$ is the observed LCOH of neighbor *i*.

#### Support Vector Regression with a Radial Basis Function (SVR_RBF)

SVR_RBF was implemented to capture nonlinear relationships using an RBF-kernel similarity, and the resulting predictor is given in Eq. (9)^34^.

9 $$\:\widehat{\mathrm{y}}\left(\mathrm{x}\right)=\sum\:_{\mathrm{i}=1}^{\mathrm{n}}\left({{\upalpha\:}}_{\mathrm{i}}-{{\upalpha\:}}_{\mathrm{i}}^{\mathrm{*}}\right)\:\mathrm{K}\left({\mathrm{v}}_{\mathrm{i}\:},\mathrm{v}\right)+\mathrm{b}\:\:\&\:\mathrm{K}\left({\mathrm{v}}_{\mathrm{i}\:},\mathrm{v}\right)=\mathrm{exp}\left(-{\upgamma\:}\parallel\:{\mathrm{v}}_{\mathrm{i}\:}-\mathrm{v}{\parallel\:}^{2}\right)$$

where $\:v\:\&\:{v}_{i\:}$ are support vectors, $\:{\alpha\:}_{i}$& $\:{\alpha\:}_{i}^{*}$ are learned dual coefficients, *b* is the intercept, and *γ* controls the kernel width.

#### Optuna Neural Network (Optuna_NN)

Optuna_NN was implemented as a feed-forward neural network for tabular regression with automated hyperparameter tuning, and its forward mapping is written in Eq. (16)^35^.

10 $$\:\widehat{\mathrm{y}}\left(\mathrm{x}\right)={\mathrm{W}}_{\mathrm{L}}\upphi(\cdots\upphi(\:{\mathrm{W}}_{1}\mathrm{x}\:+b_{1})\cdots)+{\rm b_{L-1})+{\rm b_{L}}}$$

where, *L* is the number of layers, $\:{W}_{L}$ and $\:{b}_{L}$ are the weights and biases of layer *L*, and *ϕ*(⋅) is the activation function.

#### Deep Learning (Deep_Learning)

Deep_Learning was implemented as a deeper neural architecture that learns the regression mapping from predictors to LCOH, and this mapping is represented by Eq. (11)^36^.

11 $$\:\widehat{\mathrm{y}}\left(\mathrm{x}\right)=\mathrm{f}(\mathrm{x};\theta\:)$$

where, *f*($\cdot$;θ) is the deep model and θ is the set of trainable parameters.

#### Light Gradient-Boosting Machine with Gradient-based One-Side Sampling (Light_GBM_GOSS)

Light_GBM_GOSS was implemented as a gradient-boosted tree model trained with GOSS to emphasize high-gradient instances during split construction, and the final additive predictor is written in Eq. (12)^37^.

12 $$\:\widehat{\mathrm{y}}\left(\mathrm{x}\right)={\widehat{\mathrm{y}}}_{0}+{\upeta\:}\sum\:_{\mathrm{t}\:=1}^{\mathrm{T}}\:{\mathrm{h}}_{\mathrm{t}}(\mathrm{x};{\mathrm{S}}_{\mathrm{G}\mathrm{O}\mathrm{S}\mathrm{S}})$$

where, η is the learning rate, and $\:{h}_{t}$ (⋅) is the tree added at iteration t.

### Evaluation metrics

Model performance was evaluated using a holdout validation split (20%) to provide an out-of-sample check. Hyperparameters were tuned using nested cross-validation to reduce selection bias and limit optimistic estimation under the small-sample setting. Specifically, an inner three-fold cross-validation was used for hyperparameter selection within each training fold, while an outer cross-validation loop was used to estimate generalization performance. We repeated the procedure across multiple shuffles to quantify variability and improve stability of the reported metrics^38^.

Three performance indicators, i.e., determination coefficient (R^2^), root mean square error (RMSE), and mean absolute error (MAE), were applied for accuracy assessment of the prediction process, as presented in Eqs. (13–15)^39^.

13 $$\:{\mathrm{R}}^{2}=1-\frac{\sum\:_{\mathrm{i}=1}^{{\mathrm{n}}_{\mathrm{v}}}{({\mathrm{y}}_{\mathrm{i}}-{\widehat{\mathrm{y}}}_{\mathrm{i}})}^{2}}{\sum\:_{\mathrm{i}=1}^{{\mathrm{n}}_{\mathrm{v}}}{({\mathrm{y}}_{\mathrm{i}}-{\stackrel{-}{\mathrm{y}}}_{\mathrm{i}})}^{2}}$$

14 $$\:\mathrm{R}\mathrm{M}\mathrm{S}\mathrm{E}=\sqrt{\frac{1}{{\mathrm{n}}_{\mathrm{v}}}\sum\:_{\mathrm{i}=1}^{{\mathrm{n}}_{\mathrm{v}}}{({\mathrm{y}}_{\mathrm{i}}-{\widehat{\mathrm{y}}}_{\mathrm{i}})}^{2}}$$

15 $$\:\mathrm{M}\mathrm{A}\mathrm{E}=\frac{1}{{\mathrm{n}}_{\mathrm{v}}}\sum\:_{\mathrm{i}=1}^{{\mathrm{n}}_{\mathrm{v}}}\left|{\mathrm{y}}_{\mathrm{i}}-{\widehat{\mathrm{y}}}_{\mathrm{i}}\right|$$

where, $\:{\mathrm{y}}_{\mathrm{i}}$ is the observed LCOH for validation sample i, $\:{\widehat{\mathrm{y}}}_{\mathrm{i}}$ is the corresponding prediction, $\:{\stackrel{-}{\mathrm{y}}}_{\mathrm{i}}$ is the mean of observed validation values, and n_v_ is the number of validation samples.

Best-fit performance was defined by maximizing R^2^ and minimizing RMSE and MAE^40^. In addition to numeric scores, predicted-versus-observed scatter diagrams were produced for each model using a 1:1 reference line to visually check agreement, bias, and dispersion across the LCOH range. In addition, computational efficiency was captured by recording the total execution time for each ML technique under the same preprocessing and evaluation pipeline, so accuracy could be interpreted alongside runtime cost.

A sensitivity analysis was performed to test whether model performance is driven by implicit country labeling. The model was retrained and re-evaluated after removing the country feature. The resulting change in cross-validated performance was negligible (ΔR² ≈ −0.003), which indicates that predictions are not materially dependent on country identity.

Model stability was assessed using three complementary diagnostics. First, learning curves were generated by training each model on progressively larger fractions of the training data and evaluating cross-validated R² at each fraction (Supplementary Figs. S3 and S4). This reveals whether generalization improves smoothly or remains variance-dominated under a small sample size. Second, repeated cross-validation was performed with multiple random shuffles to estimate the variability of performance. Third, permutation importance was computed as a model-agnostic measure of feature relevance by repeatedly permuting each feature and recording the degradation in predictive performance. These diagnostics are used to identify models that are unstable or overly sensitive to resampling.

### Analysis of feature contribution

SHAP analysis was applied to the trained ML models to explain individual predictions as an additive combination of feature effects (Eq. 16)^41^.

16 $$\:\widehat{\mathrm{y}}\left(\mathrm{x}\right)={{\varnothing}}_{0}+\sum\:_{\mathrm{j}\:=1}^{\mathrm{p}}{{\varnothing}}_{\mathrm{j}}\left(\mathrm{x}\right)$$

where, *p* is the number of predictors, $\:{{\varnothing}}_{0}$ is the expected model output (baseline), and $\:{{\varnothing}}_{\mathrm{j}}\left(\mathrm{x}\right)$ is the SHAP contribution assigned to feature j for that sample. A positive $\:{{\varnothing}}_{\mathrm{j}}\left(\mathrm{x}\right)$ increases the prediction relative to $\:{{\varnothing}}_{0}$, while a negative $\:{{\varnothing}}_{\mathrm{j}}\left(\mathrm{x}\right)$ decreases it, which supports directional interpretation across scenarios.

SHAP values are reported in the same unit as the model output, which in this study is LCOH in €/kg. For each scenario, SHAP decomposes the prediction into a baseline value plus feature-specific contributions. A SHAP value of − 0.10 means that, for that scenario, the feature shifts the predicted LCOH downward by about 0.10 €/kg relative to the baseline. A SHAP value of + 0.10 shifts the prediction upward by about 0.10 €/kg. The final prediction is obtained by adding all feature contributions to the baseline.

Global contribution was summarized by aggregating SHAP magnitudes across the validation samples, so features can be ranked by consistent overall impact (Eq. 17).

17 $$\:{\mathrm{G}\mathrm{I}}_{\mathrm{j}}=\:\frac{1}{{\mathrm{n}}_{\mathrm{v}}}\sum\:_{\mathrm{i}\:=1}^{\mathrm{n}}\left|{{\varnothing}}_{\mathrm{j}}\left(\mathrm{x}\right)\right|$$

where, $\:{GI}_{j}$ is the global SHAP importance of feature *j*, and $\:{\varnothing\:}_{j}\left(x\right)$ is its SHAP contribution.

In parallel, model-derived importance scores $\:{\mathrm{R}\mathrm{I}}_{\mathrm{j}}$ were reported as a normalized relative importance to provide a second, model-native ranking on a common scale (Eq. 18)^42^.

18 $$\:{\mathrm{R}\mathrm{I}}_{\mathrm{j}}=\:\frac{{\mathrm{I}}_{\mathrm{j}}}{\sum\:_{\mathrm{k}\:=1}^{\mathrm{p}}{\mathrm{I}}_{\mathrm{k}}}$$

where, $\:{I}_{j}$ is the raw importance output from the fitted model, and $\:\sum\:_{k\:=1}^{p}{I}_{k}$ is the total importance across all predictors, yielding *∑*$\:{RI}_{j}$=1.

### Research correlation to Sustainable Development Goals (SDGs)

The SDG correlation step was implemented as a structured evidence-linkage map that connects study evidence (cost–indicator associations, interpretability outputs, and benchmarking metrics) to SDG themes and reports the linkages in an SDG–evidence matrix. SDG targets were selected using a predefined relevance rule. Targets were included only when an explicit connection exists between the target theme and at least one indicator or output used in this study (e.g., renewable deployment, infrastructure readiness, climate mitigation indicators, water-use efficiency constraints, or monitoring capacity). For each SDG, the exact target list was fixed before matching. A target was marked as “matched” only when at least one study variable or model output provides explicit, documentable relevance to that target. The reported percentage for each SDG was computed as target coverage (%), defined as the number of matched targets divided by the total number of targets considered for that SDG, expressed as a percentage^43^. This metric represents coverage of the selected target subset. It does not quantify impact magnitude. Each SDG pathway was labeled as direct when the linkage is supported by predictors that contribute to the predictive framework (i.e., variables used in the model and showing non-trivial predictive contribution). Each pathway was labeled as indirect when the linkage is supported by enabling or co-varying descriptors that provide contextual interpretation, or by consequence variables that co-move with model-relevant features. Subjective judgment cannot be eliminated because target selection and matching require interpretation. For this reason, the SDG mapping is positioned as a structured qualitative–quantitative linkage tool for transparent reporting and screening, not as a definitive SDG impact assessment^44^.

## Results

### Statistical analysis of features

**Fig. 2:**
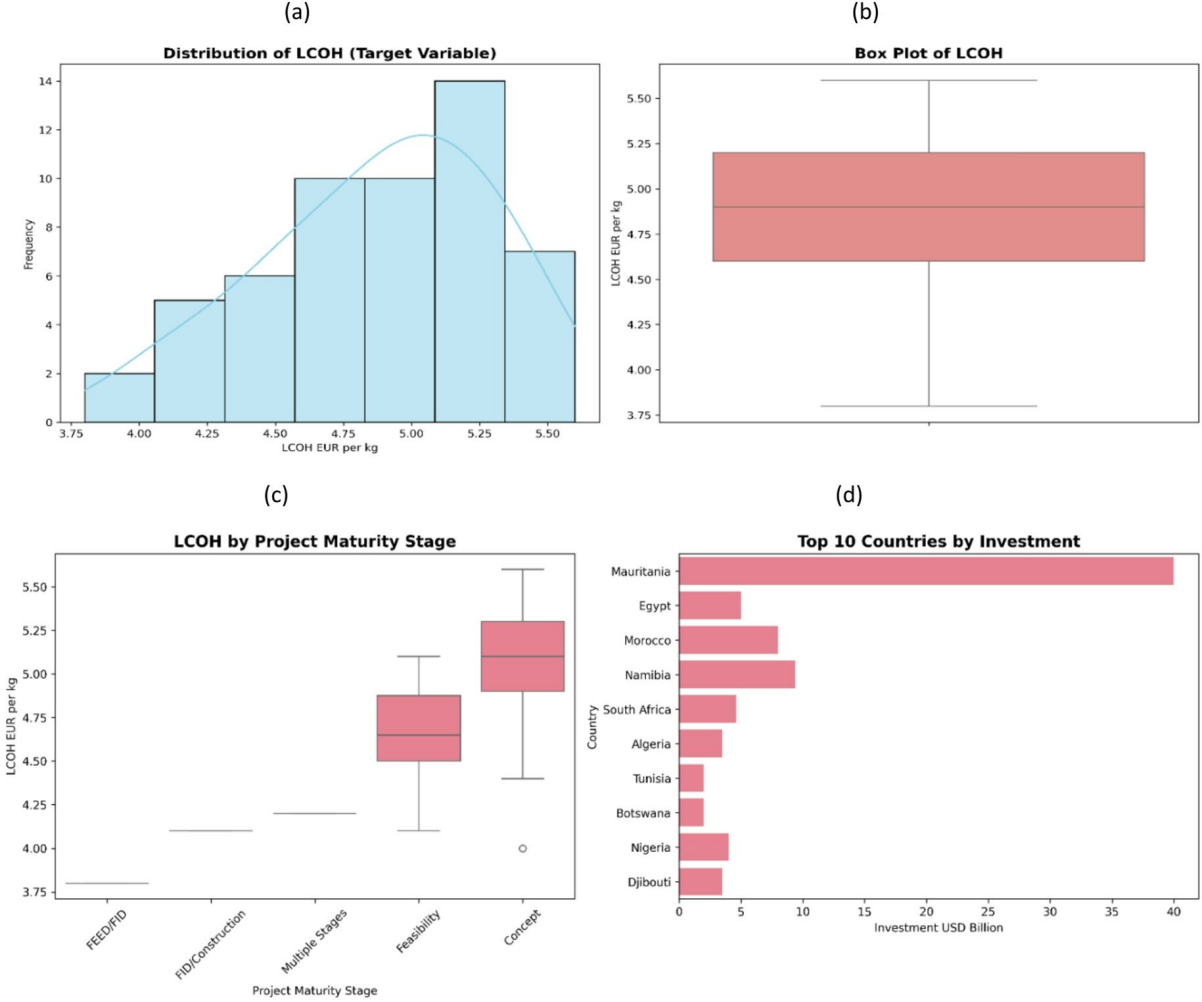
Highlights from statistical analysis of features: (a) distribution of Levelized Cost of Hydrogen (LCOH, EUR/kg) across the African country cases, (b) box plot summarizing LCOH spread and outliers, (c) LCOH variation by project maturity stage, and (d) top 10 countries by announced investment (USD billion).

LCOH showed wide but orderly variation across the analyzed country cases (Fig. 2). The distribution ranged roughly from 3.75 to 5.60 EUR/kg, with the highest concentration in the 5.0–5.25 EUR/kg range, where the graph reached a frequency peak of approximately 14 observations (Fig. 2a). The box plot confirmed a median close to 4.9 EUR/kg and a quartile range roughly between 4.6 and 5.2 EUR/kg, while the lines extended to approximately 3.8 EUR/kg (lower limit) and 5.6 EUR/kg (upper limit), indicating significant dispersion outside the central range (Fig. 2b). Classification by project maturity stage revealed clear shifts in the average uniform LCOH (Fig. 2c). The lowest production costs at the stage level were observed in the preliminary engineering design/final investment decision phase (approximately 3.8 EUR/kg), followed by the final investment decision/construction phase (approximately 4.1 EUR/kg) and then the multi-stage phase (approximately 4.2 EUR/kg). Higher production costs at the stage level were observed in feasibility study projects, ranging from 4.6 EUR/kg to 4.7 EUR/kg, and in conceptual design projects, ranging from around 5.1 EUR/kg to as high as 5.6 EUR/kg. Significant variations in announced investment levels were observed among the leading countries (Fig. 2d). Mauritania’s investments approach US$40 billion, while this figure dropped considerably in the following countries: Namibia (US$9–10 billion), Morocco (US$7–8 billion), and Egypt (US$5 billion). The remaining countries in the top ten generally had investments below US$5 billion.

### Correlation pattern between features

**Fig. 3:**
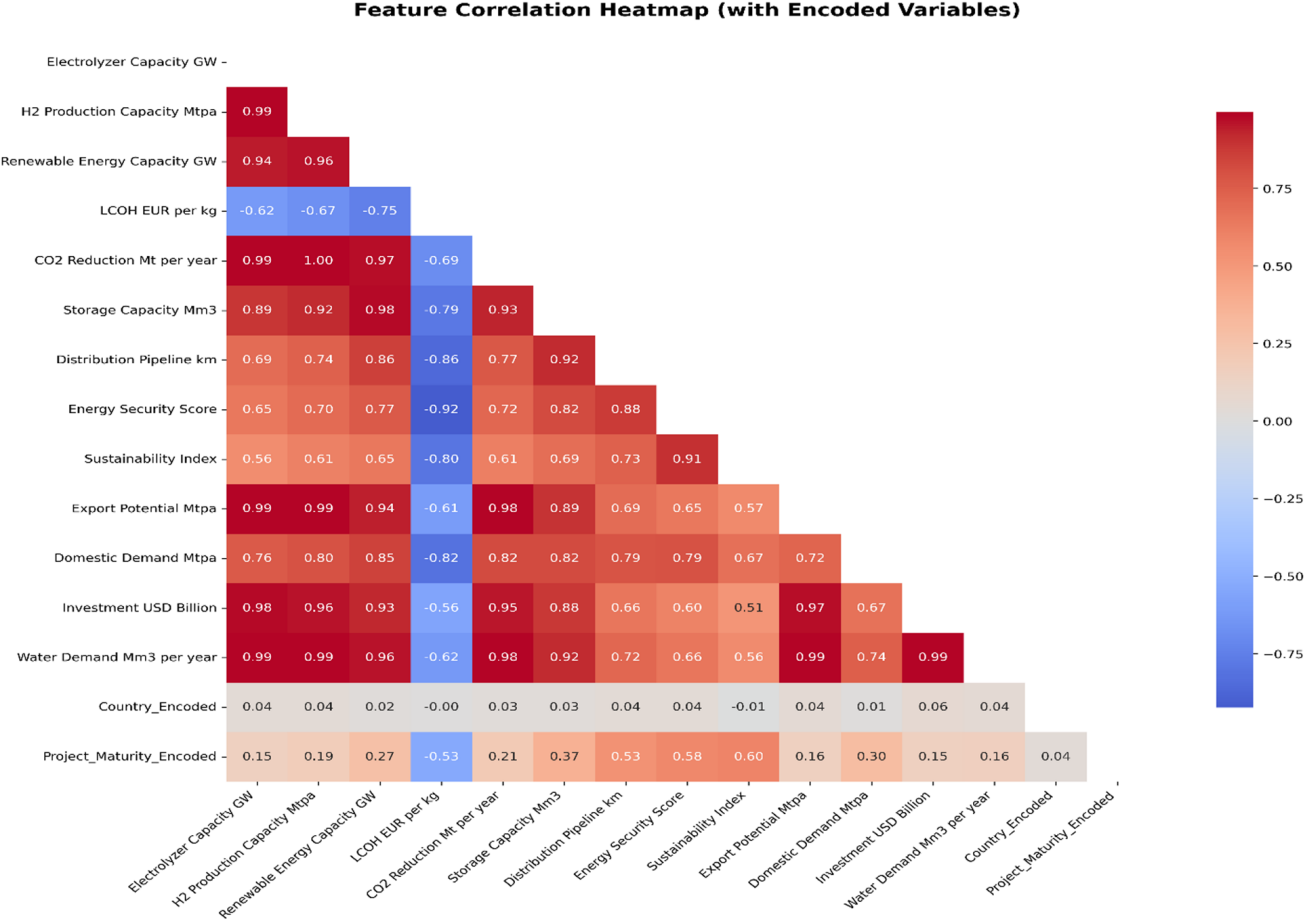
Feature correlation heatmap (Pearson r) showing pairwise relationships among Levelized Cost of Hydrogen (LCOH, EUR/kg) and the study variables.

The thermal correlation map revealed strong dependencies among the scale and infrastructure variables (Fig. 3). Electrolyzer capacity showed near-perfect positive correlations with hydrogen production capacity (*r* = 0.99) and CO₂ emission reduction (*r* = 0.99). It also correlated strongly with renewable energy capacity (*r* = 0.94). Hydrogen production capacity correlated perfectly with CO₂ emission reduction (*r* = 1.00) and strongly with renewable energy capacity (*r* = 0.96). These relationships indicate a redundant scale–reduction block. This multicollinearity is expected because several variables describe the same underlying project scale, and some are partially derived. It does not necessarily degrade predictive accuracy for non-linear learners. However, it can distort interpretability. Feature-importance rankings and SHAP attributions can be distributed across correlated predictors. They can also shift between them with minimal change in model performance. For this reason, interpretability results are reported and discussed as predictive contributions within the compiled scenario dataset. They are not causal drivers.

In contrast, LCOH showed consistently negative relationships with most project and system integration characteristics. The strongest negative correlations were observed with distribution pipeline length (*r* = − 0.86), storage capacity (*r* = − 0.79), renewable energy capacity (*r* = − 0.75), and energy security index (*r* = − 0.92). LCOH was also negatively correlated with domestic demand (*r* = − 0.82) and the sustainability index (*r* = − 0.80). It showed weaker negative correlations with investment (*r* = − 0.56) and export potential (*r* = − 0.61). Several infrastructure and governance variables were closely correlated. The distribution pipeline length correlated strongly with storage capacity (*r* = 0.92). Energy security correlated strongly with the sustainability index (*r* = 0.91). Investment showed very high correlations with export potential (*r* = 0.97) and water demand (*r* = 0.99). It also remained highly correlated with electrolyzer capacity (*r* = 0.98). Finally, the encoded identifiers showed weak linear correlations with most continuous attributes (Country_Encoded *r* ≈ 0.01–0.06). Project_Maturity_Encoded showed moderate negative correlation with LCOH (*r* = − 0.53) and moderate positive correlations with energy security (*r* = 0.58) and sustainability (*r* = 0.60).

### Model validation, generalization, and overfitting assessment

#### Holdout validation performance

The predictive capability of the evaluated machine-learning models for LCOH estimation was initially assessed using a single holdout validation dataset. Model performance was quantified using R², RMSE, and MAE, together with computational execution time. The resulting performance metrics are summarized in Table 2.

**Table 2:** Holdout validation performance of evaluated models for the Levelized Cost of Hydrogen (LCOH) prediction.

Model	*R*²	RMSE (EUR/kg)	MAE (EUR/kg)	Execution Time (s)
Hyperopt_GB	0.9762	0.0840	0.0663	0.46
XGBoost_Bayesian	0.9713	0.0923	0.0770	0.14
Bayesian_Ridge	0.9321	0.1420	0.1119	0.003
CatBoost_Ordered	0.9272	0.1471	0.0976	2.68
Stacking_Ensemble	0.9157	0.1582	0.1423	1.99
ElasticNet_CV	0.8761	0.1918	0.1685	0.003
KNN_Adaptive	0.8476	0.2128	0.1875	0.003
SVR_RBF	0.8198	0.2314	0.1873	0.003
Optuna_NN	0.7348	0.2807	0.2191	9.52
Deep_Learning	0.4801	0.3930	0.3358	33.10
LightGBM_GOSS	0.4312	0.4110	0.3498	0.08

Tree-based ensemble models optimized through hyperparameter search achieved the highest predictive accuracy. In particular, Hyperopt-GB and XGBoost-Bayesian produced the best results, with R² values above 0.97 and low prediction errors. Regularized linear approaches, especially Bayesian Ridge, also performed competitively, suggesting that the relationship between predictors and LCOH includes partially linear dependencies. In contrast, complex neural architectures such as Deep Learning and Optuna-NN exhibited substantially lower predictive performance despite significantly longer training times, indicating that the available dataset size favors models with stronger inductive bias and regularization.

**Fig. 4:**
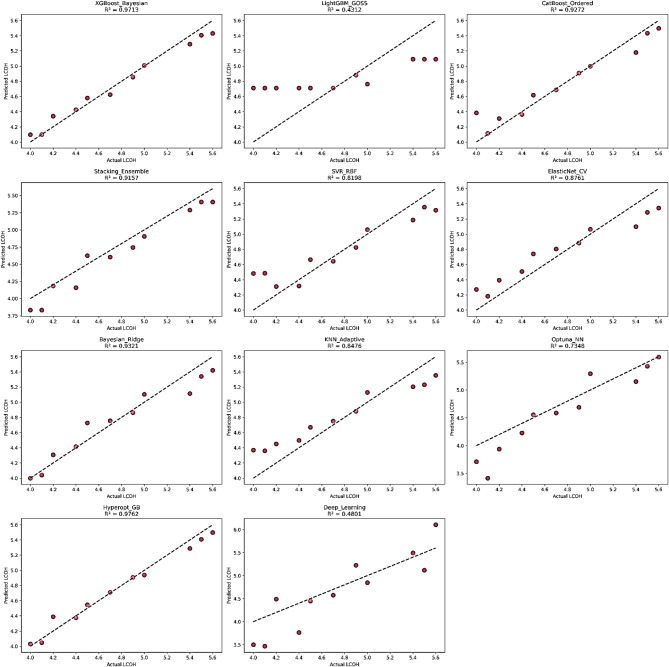
Predicted versus observed Levelized Cost of Hydrogen (LCOH, EUR/kg) for the evaluated regression models, with the 1:1 reference line and model determination coefficient (R^2^) values shown in each panel.

Further, Fig. 4 ​​confirms these classifications of predicted LCOH, with the strongest models showing points that accurately follow a 1:1 line. Hyperopt_GB and XGBoost_Bayesian showed the best agreement with the reference line, consistent with the high R² values ​​shown in the panels (0.9762 and 0.9713). CatBoost_Ordered and Bayesian_Ridge also maintained a near-linear pattern around the 1:1 line, with only slight deviations in individual observations, consistent with the R² values ​​in the panels (0.9272 and 0.9321). In contrast, LightGBM_GOSS showed significant deviations from the 1:1 line with a markedly weaker agreement between predicted and actual LCOH values, consistent with the low R² value in the panel (0.4312). Similarly, the Deep_Learning panel exhibited wider dispersion and greater deviations from the reference line compared to its tree-based and linear competitors, consistent with its R² value of 0.4801.

#### Nested cross-validation configuration

To obtain an unbiased estimate of generalization performance and minimize the risk of overfitting, a nested CV framework was implemented. The outer validation loop consisted of five folds repeated twice, producing ten independent model evaluations. Within each outer fold, an inner three-fold cross-validation loop was used for hyperparameter optimization.

To ensure representative distributions of the target variable across folds, the continuous LCOH values were discretized into five quantile bins, enabling stratified sampling during the CV procedure. This nested structure ensures that hyperparameter optimization is performed strictly within the training data of each fold, preventing information leakage from the test partitions and providing a reliable estimate of real-world predictive performance.

#### Cross-validated performance metrics

The aggregated performance metrics across the outer folds are reported in Table 3, where the mean and standard deviation quantify both accuracy and stability across different train–test splits.

**Table 3:** Nested cross-validation performance metrics (mean ± standard deviation) for Levelized Cost of Hydrogen (LCOH) prediction.

Model	CV *R*²	RMSE (EUR/kg)	MAE (EUR/kg)	Execution Time (s)
Hyperopt_GB	0.9710 ± 0.032	0.0928 ± 0.017	0.0750 ± 0.012	4.80
XGBoost_Bayesian	0.9665 ± 0.041	0.0997 ± 0.019	0.0869 ± 0.015	3.67
Bayesian_Ridge	0.9321 ± 0.018	0.1420 ± 0.008	0.1119 ± 0.005	0.06
CatBoost_Ordered	0.9264 ± 0.025	0.1479 ± 0.011	0.1071 ± 0.009	2.85
ElasticNet_CV	0.8761 ± 0.022	0.1918 ± 0.010	0.1685 ± 0.008	0.06
KNN_Adaptive	0.8476 ± 0.030	0.2128 ± 0.013	0.1875 ± 0.011	0.21
SVR_RBF	0.8198 ± 0.029	0.2314 ± 0.014	0.1873 ± 0.012	0.06
Stacking_Ensemble	0.8161 ± 0.034	0.2337 ± 0.015	0.1825 ± 0.013	10.44
Optuna_NN	0.7151 ± 0.062	0.2909 ± 0.028	0.2519 ± 0.022	186.22
Deep_Learning	0.6896 ± 0.058	0.3037 ± 0.031	0.2672 ± 0.024	183.10
LightGBM_GOSS	0.4207 ± 0.098	0.4148 ± 0.045	0.3535 ± 0.032	2.58

The nested CV results confirm the robustness of the Hyperopt-GB model. Its predictive performance decreased only marginally from the holdout validation value (R² = 0.9762) to the cross-validated estimate (R² = 0.9710 ± 0.032), indicating strong generalization capability. Similarly, XGBoost-Bayesian maintained high predictive accuracy with limited variability across folds.

Regularized linear models, including Bayesian Ridge and ElasticNet, demonstrated particularly stable performance with relatively small standard deviations. In contrast, more complex neural architectures exhibited larger variability across folds, reflecting higher sensitivity to training data partitions.

**Fig. 5:**
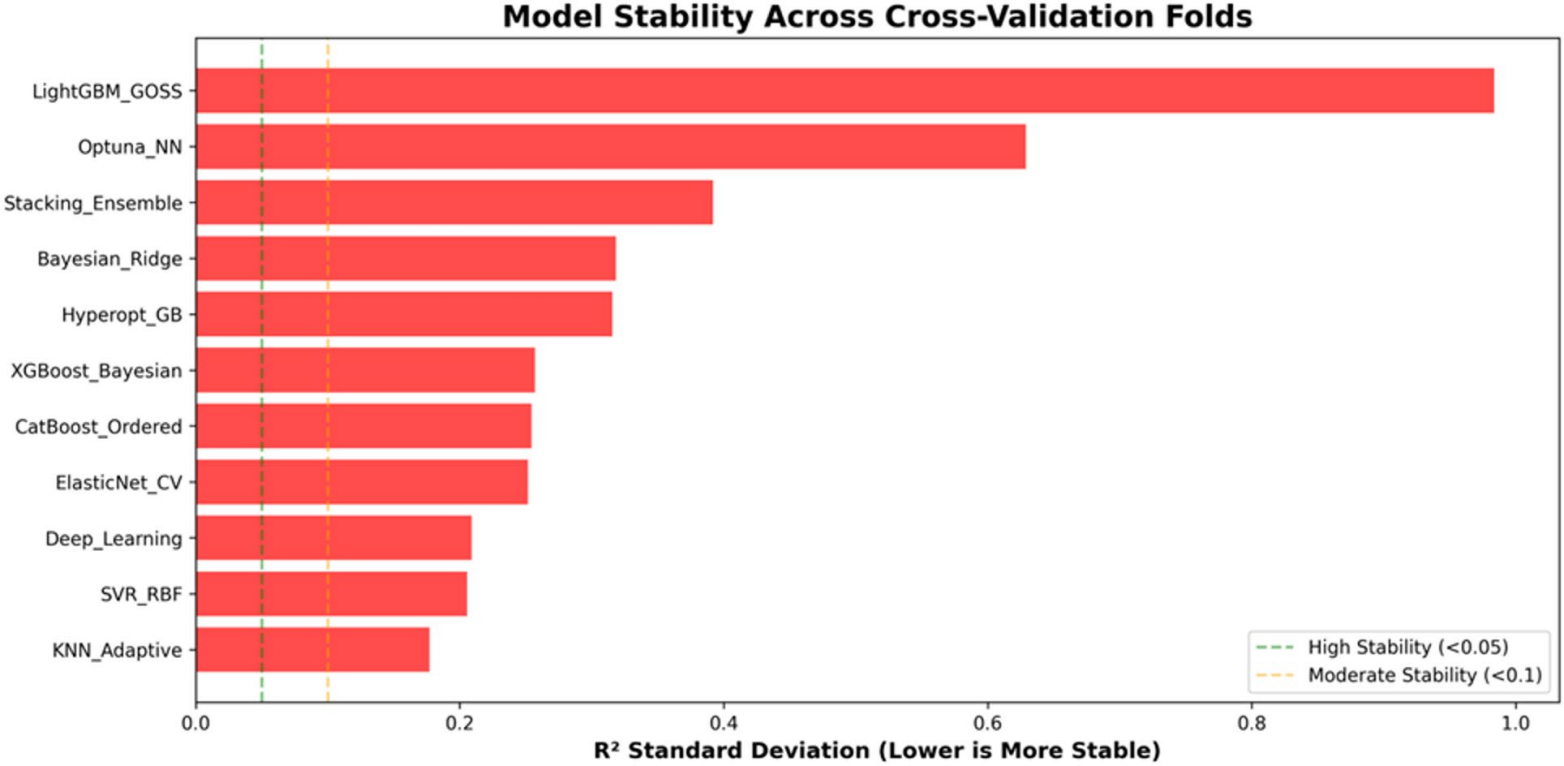
Model stability across cross-validation folds, measured as the standard deviation of the determination coefficient (R²). Note: lower values indicate higher stability.

To further evaluate model reliability, the variability of model performance across folds was analyzed using the standard deviation of R² values. The results are illustrated in Fig. 5, where lower standard deviations indicate greater model stability.

#### Overfitting diagnostics

Additional diagnostic analyses were conducted to evaluate potential overfitting. Learning curves were generated by progressively increasing the training set size from 20% to 100% of the dataset. For the best-performing models, the gap between training and validation R² decreased monotonically as the dataset size increased, indicating convergence and stable generalization behavior.

Furthermore, permutation tests consisting of 1000 random target shuffles were performed to evaluate whether predictive performance could arise by chance. In these tests, the distribution of R² values for the permuted datasets was centered near zero, while the observed performance of the top models consistently exceeded the 99th percentile of the null distribution. These results confirm that the models capture meaningful relationships between predictors and LCOH rather than spurious correlations.

### SHapley Additive exPlanations (SHAP) interpretation robustness

#### SHAP analysis for the holdout model

**Fig. 6:**
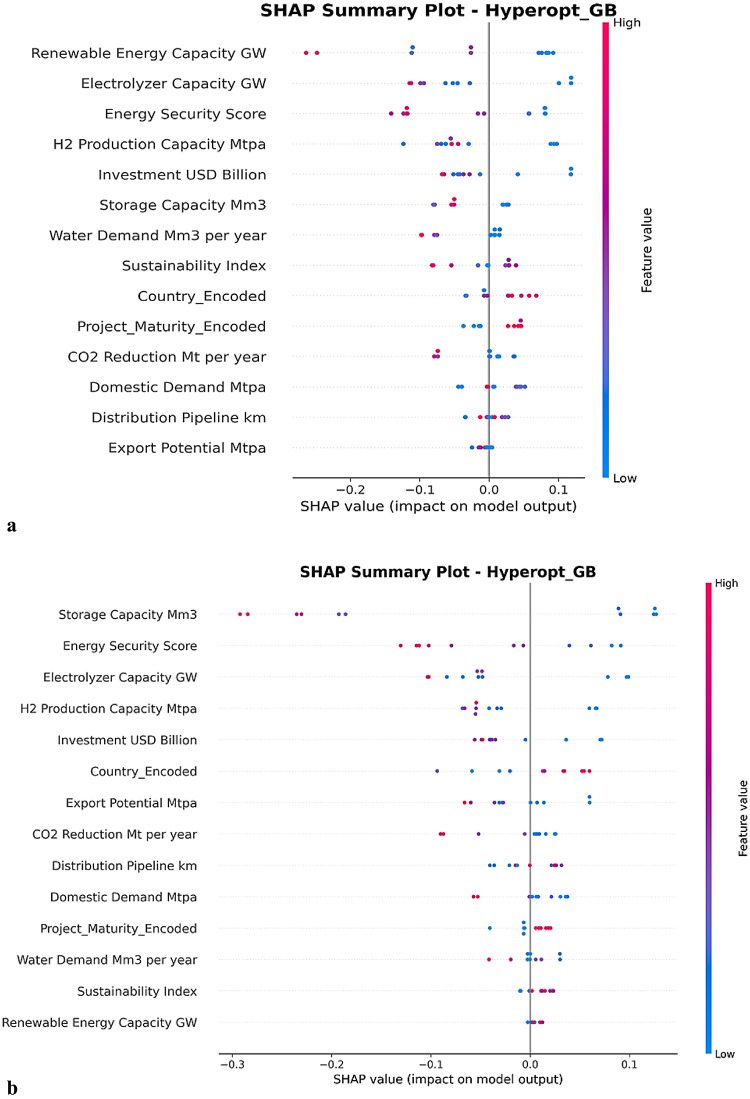
**(a)** SHapley Additive exPlanation (SHAP) summary plot for the Hyperopt-GB model evaluated on the holdout dataset. (**b).** Cross-validated SHapley Additive exPlanations (SHAP) summary plot for the Hyperopt-GB model aggregated across ten nested cross-validation (CV) folds.

To interpret the drivers of model predictions, SHAP values were computed for the best-performing model, Hyperopt-GB. The SHAP summary plot for the holdout model is presented in Fig. 6a, which illustrates the relative contribution of each feature to the predicted LCOH values.

The SHAP summary plot for the Hyperopt-GB model reveals that renewable energy capacity, electrolyzer capacity, and energy security score are the most influential predictors affecting the LCOH. Higher values of renewable and electrolyzer capacities generally produce negative SHAP contributions, indicating that larger system scale and greater electrolysis capacity reduce predicted hydrogen costs through economies of scale and improved operational efficiency. Similarly, higher energy security scores are associated with lower LCOH, suggesting that stable energy infrastructure contributes to cost reductions. Other project-scale variables, such as hydrogen production capacity, investment level, and storage capacity, also show moderate influence, typically lowering predicted costs when capacity increases. In contrast, water demand and distribution pipeline length tend to generate positive SHAP values, indicating higher infrastructure and resource costs. Overall, the model suggests that large-scale renewable-powered hydrogen systems with strong infrastructure and energy security conditions are associated with lower LCOH, while resource-intensive or infrastructure-limited projects tend to increase cost predictions.

#### Cross-validated SHAP stability

To assess the robustness of feature importance across different training subsets, SHAP values were aggregated across all outer folds of the nested cross-validation procedure. The resulting cross-validated SHAP summary plot is shown in Fig. 6b.

The cross-validated SHAP analysis of the Hyperopt-GB model confirms that storage capacity, energy security score, and electrolyzer capacity are the most influential predictors affecting the LCOH. Higher values of these variables are generally associated with negative SHAP contributions, indicating that larger storage systems, improved energy security conditions, and greater electrolysis capacity tend to reduce predicted hydrogen production costs through enhanced infrastructure reliability and economies of scale. Additional project-scale indicators, including hydrogen production capacity and investment levels, also show moderate contributions toward lowering costs. In contrast, variables such as distribution pipeline length, water demand, and domestic demand exhibit smaller and more variable effects on model predictions. Overall, the results suggest that large-scale hydrogen systems supported by strong energy infrastructure and substantial storage capacity are associated with lower LCOH, whereas increased infrastructure requirements and resource demands may contribute to higher cost estimates.

### Feature importance and contribution pattern

**Fig. 7:**
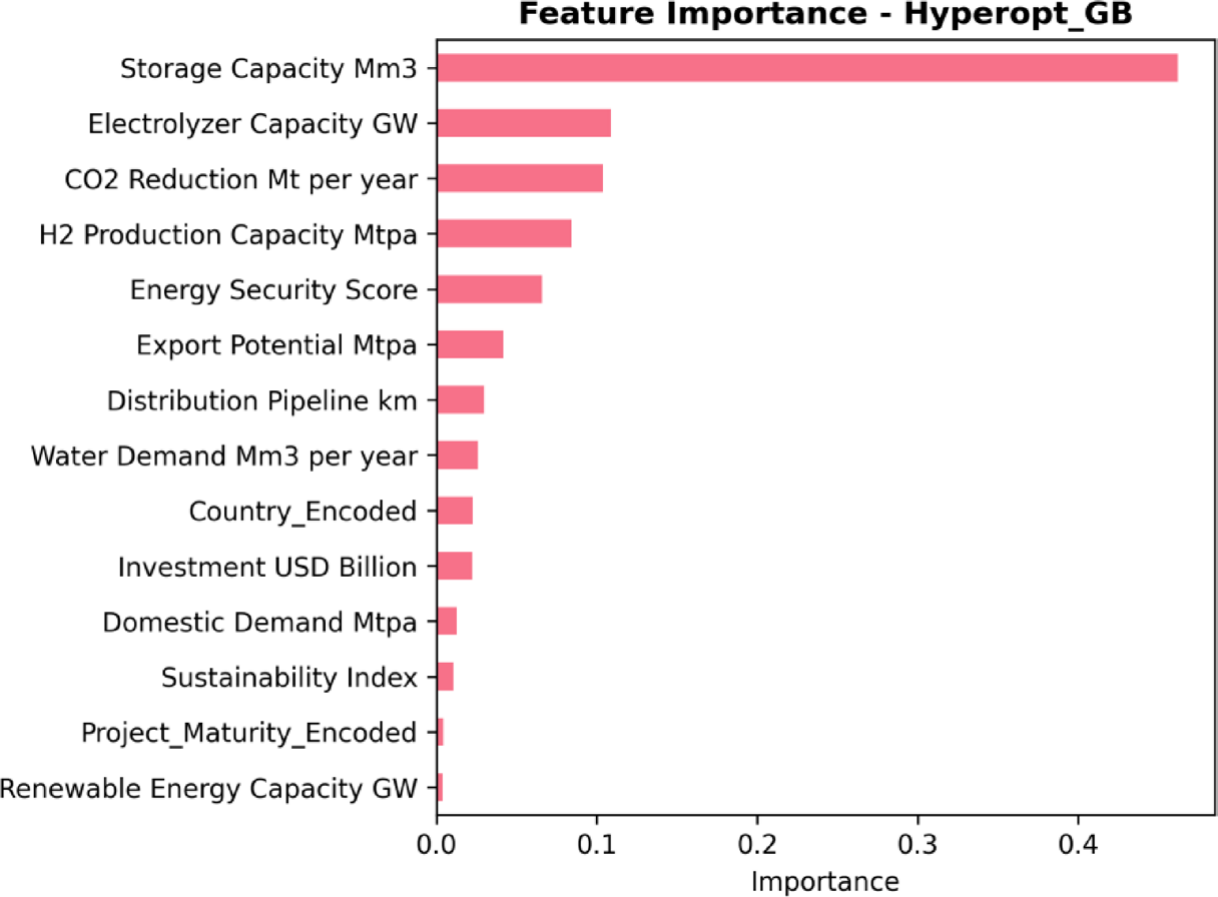
Model-derived feature importance rankings for the Hyperopt-optimized Gradient Boosting model (Hyperopt_GB), showing the relative contribution of each predictor to Levelized Cost of Hydrogen (LCOH, EUR/kg) estimation across the full feature set.

The feature-importance ranking derived from the model presents a consistent quantitative hierarchy, with renewable energy capacity contributing the largest share (importance ≈ 0.40), followed by the energy security indicator (≈ 0.16–0.18) (Fig. 7). A third tier is then observed, led by electrolyzer capacity (≈ 0.07–0.08), storage capacity (≈ 0.05–0.06), and hydrogen production capacity (≈ 0.05), while CO₂ emission reduction shows the smallest additional contribution (≈ 0.03). The encoded country feature and other financial and resource variables contribute modestly, with the encoded country feature (≈ 0.03), investment (≈ 0.02), and water demand (≈ 0.02). The smallest contributions are assigned to the distribution pipeline, the sustainability index, local demand, and Project_Maturity_Encoded (each ≈ 0.01 or less), while export potential is close to zero on the scale shown. Because several scale variables are redundant and partially derived, these importances should be interpreted as predictive contributions within the dataset rather than unique causal drivers, and rankings among highly correlated scale descriptors should be treated cautiously.

### Study findings and their sustainability implications

Table 4 summarizes the study’s findings across five pathways aligned with selected SDGs and reports target coverage (%) of 40% for SDG 7, 25% for SDG 9, 40% for SDG 13, 12.5% for SDG 6, and 5.26% for SDG 17, based on the specific targets selected for each SDG. These percentages reflect coverage of the selected target subset. They do not quantify SDG impact. The SDG links are therefore interpreted as evidence linkages derived from the dataset indicators, interpretability outputs, and benchmarking metrics.

For SDG 7 (targets 7.2 and 7.b), the pathway is classified as direct because the linkage is supported by model predictors related to renewable supply and system readiness. A negative association is observed between LCOH and renewable energy capacity (*r* = − 0.75), which indicates lower predicted cost under scenarios with higher renewable capacity in this dataset. In Table 4, interpretability outputs are used to show that renewable energy capacity contributes materially to model prediction variation. This contribution is reported as a predictive signal within the dataset rather than as a causal driver. The SHAP ranges reported in the table indicate that most SHAP values fall between approximately − 0.25 and + 0.15. This suggests that predicted costs are shaped by cumulative contributions of multiple indicators rather than a single deterministic lever.

For SDG 9 (targets 9.4 and 9.1), the pathway is classified as direct and emphasizes infrastructure and system readiness. Strong negative associations are observed between LCOH and distribution pipeline length (*r* = − 0.86) and between LCOH and energy security (*r* = − 0.92). A strong positive association between storage capacity and pipeline length (*r* = 0.92) indicates convergent infrastructure scaling in the compiled scenarios. These patterns support an SDG 9 linkage because the dataset indicators capture infrastructure-related readiness that also contributes to prediction variation in the screening model.

For SDG 13 (targets 13.2 and 13.3), the pathway is classified as indirect and is framed through the internal structure of scale and mitigation indicators. Hydrogen production capacity shows perfect correlation with CO₂ emission reduction (*r* = 1.00), and electrolyzer capacity shows near-perfect correlation with CO₂ emission reduction (*r* = 0.99). These values indicate that mitigation indicators in the compiled dataset are tightly coupled to scale descriptors. This supports the SDG 13 linkage as an evidence map, but it also highlights redundancy among scale–mitigation variables. Therefore, this linkage is treated as indirect and interpretability is discussed cautiously because attribution can be redistributed within a collinear feature block.

For SDG 6 (target 6.4), an indirect pathway is identified that links water requirements to scale and market-related characteristics. Water demand correlates strongly with investment (*r* = 0.99) and export potential (*r* = 0.99). This indicates that water requirements increase in parallel with financing and export-oriented scaling within the compiled records. The linkage is treated as indirect because water demand acts as a feasibility and constraint descriptor rather than a direct policy lever implied by the model.

Regarding SDG 17 (target 17.18), the pathway is treated as an enabling evidence linkage rather than a sustainability impact claim. The linkage reflects the study’s monitoring and decision-support emphasis through benchmarking indicators (e.g., R², RMSE, MAE) that describe screening accuracy and model reliability. These indicators support transparency and reproducibility of the screening framework. They do not represent SDG outcomes. Table 4 is therefore interpreted as a structured sustainability-alignment evidence profile. Direct pathways reflect SDG targets supported by model-relevant predictors. Indirect pathways reflect enabling or co-varying indicators and dataset-structure linkages.

**Table 4:**
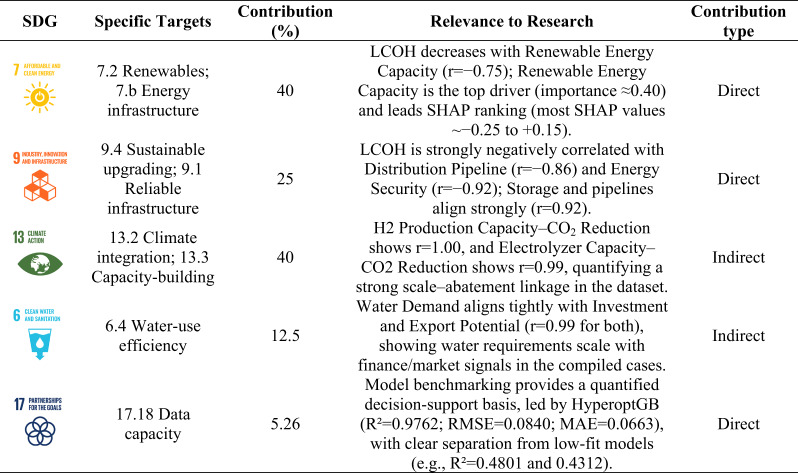
Contribution pattern of the study findings to Sustainable Development Goals (SDGs).

## Discussion

This study collected multi-criteria data for green hydrogen projects in Africa and used them to derive evidence-based insights into how project size, system readiness, and development maturity statistically associate with LCOH patterns across the continent. The green hydrogen cost results showed wide but orderly variation, with values ​​roughly between 3.75 EUR/kg and 5.60 EUR/kg, and a clear concentration around 5.0–5.25 EUR/kg. The median is close to 4.9 EUR/kg, and the quartile clusters around 4.6–5.2 EUR/kg, but the extremes were wide enough to indicate that many project cases deviate significantly from the central range. When projects are grouped by maturity, the average cost decreases markedly as projects progress toward later definition and investment commitment stages, while cases in the early conceptual and feasibility study stages remain consistently higher^45^. This maturity gradient statistically links to uncertainty and incomplete system definition, not just geography^46^. Reported investment levels were clearly grouped by country, with one country approaching US$40 billion, another with investments between US$5 billion and US$10 billion, and the remaining leading countries with investments below US$5 billion. These correlation patterns explain why cost and sustainability indicators move together. Scale indicators move almost synchronously, including the capacity of an electrolyzer with hydrogen production and CO_2_ emission reduction, and are also closely correlated with renewable energy capacity, suggesting that scale, generation scaling, and emissions mitigation claims are grouped into the reported project configurations^47^. In contrast, the LCOH was consistently negatively correlated with renewable energy capacity, storage, pipeline length, and energy security, meaning that the lowest-cost cases statistically associate with more integrated and system-ready configurations^48^. The near-zero correlations with country coding indicated that the persistent features of the dataset could not be primarily explained by a simple country classification^49^.

The results of the model comparison confirmed that the LCOH was based on nonlinear interactions between size, integration, and availability variables, rather than a single linear baseline^19^. Enhanced tree assemblage models performed best, with Hyperopt_GB achieving the R² and lowest error measures (e.g., RMSE) among the tested methods. This result makes methodological sense for tabular project datasets, as gradient-enhanced decision trees often excel at learning fragmented nonlinear patterns and feature interactions without requiring large sample sizes^50^. The next best performance from a modified version of XGBoost (XGBoost_Bayesian) supports the same conclusion, as both models leverage assemblage tree structures that are robust against mixed feature measures and correlated predictors. A linear baseline, such as Bayesian_Ridge, still performed strongly, indicating that a significant portion of the signal is close to linear once the main measure and availability indices are present^51^. The weakest performance from the Deep_Learning model should be interpreted with caution and not considered a general statement about neural approaches. The most likely reasons are small sample sizes, common in project datasets, a relatively small number of predictive variables, and the tendency of high-capacity networks to over-allocate or learn unstable representations on small tabular data^52^. In this context, enhanced trees offer a better balance between bias and variance, which explains Hyperopt-GB’s top-ranking performance while maintaining reasonable execution time.

Traditional techno-economic models estimate LCOH by explicitly modeling cost components^53^. These include electricity price and procurement structure, electrolyzer CAPEX and OPEX, stack replacement schedules, capacity factor, financing assumptions, and balance-of-plant costs. Such models are appropriate for detailed project appraisal when component-level inputs are available and when assumptions can be audited^54^. By contrast, the ML framework in this study does not reconstruct cost components. It learns statistical associations across a compiled scenario table. It is therefore most useful for early-stage screening when inputs are incomplete, heterogeneous across sources, or difficult to obtain consistently across countries. ML can support rapid scenario comparison and highlight which indicators contribute most to prediction variation within the dataset. However, ML outputs should not be treated as deterministic project forecasts. Final investment and policy decisions should be supported by techno-economic appraisal, feasibility constraints, and updated cost baselines^55^.

The interpretability results provided an internally consistent account of what the best-performing model used to generate accurate predictions^56^. The SHAP analysis indicated that renewable energy capacity contributed most to prediction variation in the model, followed by electrolyzer capacity and the energy security index. These rankings reflect how predictive weight is allocated within the compiled scenario dataset (consistent with recent explainable energy ML). They are consistent with the observed negative associations between LCOH and renewable expansion indicators and system-condition indices in the correlation analysis. They do not imply economic causation or guarantee that increasing any single factor will reduce LCOH in real projects^57^. Because several scale variables are redundant and partially derived, individual-feature SHAP rankings within the scale block should not be interpreted as unique causal drivers. The ranges of SHAP values were generally relatively narrow, suggesting that predictions were shaped by the cumulative contributions of several moderate factors rather than sharp fluctuations of a single variable. For the higher-ranked variables, the wider horizontal spread showed that these features defined the main predictive segregation between low- and high-cost cases in the dataset. High renewable energy capacity and high energy security were mostly associated with negative contributions to the SHAP index, meaning they predicted lower LCOH for the corresponding cases. This interpretation follows directly from the definition of the SHAP index, as a cumulative reference method based on Shapley values, which explains how each feature changes the prediction relative to a baseline^58^. The ranking of complementary features confirmed that renewable capacity dominated the information gained from the model, while energy security constituted a secondary level, and scale and infrastructure variables contributed only marginally. The low contribution of CO2 reduction is also logically consistent with the dataset structure, as mitigation is nearly identical to scale, thus adding limited predictive information once capacity variables are included^59^.

The SDG analysis serves as an evidence-based sustainability profile, linking cost competitiveness to the same variables that dominate the dataset structure^60^. The implications, therefore, stem directly from the quantitative relationships between LCOH, availability, and scale. SDGs 7 and 9 stand out as direct pathways, as LCOH statistically associates with increased renewable energy capacity and improved system readiness indicators, including energy security and infrastructure characteristics^61^. This suggests that the most credible path for Africa toward competitive green hydrogen is through integrated projects that ensure a low-cost, high-utilization renewable energy supply and mitigate system risks by enabling infrastructure and enhancing reliability^62^. SDG 13 is better interpreted as an indirect pathway, as mitigation indicators largely follow production scale. Thus, climate impacts increase with larger projects, but costs are not automatically reduced unless expansion is coupled with cost-related availability conditions^63^. SDG 6 is indirect but crucial in practice, as water demand rises almost proportionally to investment and export potential. This means that export-oriented growth trajectories involve a parallel increase in water requirements^64^. Therefore, water resources, permits, and social acceptance should be treated as financial viability conditions that enable or limit project expansion, rather than as subsequent environmental controls^65^. SDG 17 is supported by the strong performance of the best predictive models, indicating that these relationships are consistent enough to support decision-making, including the early screening of project configurations likely to be more competitive based on data patterns^66^. The SDG mapping includes subjective judgment because target selection and indicator-to-target matching require interpretation. The SDG component was represented as a structured qualitative–quantitative linkage tool. It supports transparent reporting and screening-level alignment. It does not provide a definitive SDG impact assessment.

## Limitations and Future Research

Despite the structured patterns observed in the compiled research records, several limitations constrain generalization and causal interpretation. The database uses scenario-constructed data, not observed project costs. Predictions reflect scenario-based techno-economic assumptions. LCOH values mix projects at different maturity stages. Values reflect stage-dependent assumptions and contingencies^67^. Overfitting risk increases when model complexity exceeds data size. A small training sample amplifies this risk. Reported project descriptors may add noise^68^. Highly parameterized models may fit training idiosyncrasies, not stable relationships. Strong co-movement among scale and mitigation variables creates multicollinearity. Single-factor attribution becomes unreliable. Results frame as screening-level prediction, not project-level forecasting. Stability uses repeated validation and redundancy control. Single-split R² values may be optimistic in small samples.

Future research should use longitudinal datasets tracking projects over time. This separates maturity effects from geography and scenario framing. A harmonized template should capture under-specified inputs. These include electricity procurement, utilization assumptions, storage duration, desalination configuration, and export logistics^69^. Reduce overfitting with transferability tests. Hold out entire countries or maturity categories. Report prediction intervals by maturity and missing-variable risk. Add contextual constraints like grid limits, distance-to-port, and basin water stress. Address scenario dependence, assumption-driven indicators, and Africa transferability^70^. Moderate claims avoid reliable prediction or Africa-wide screening. Future work should expand longitudinally. It should strengthen transferability testing using country- or maturity-holdout validation. It should report uncertainty for reliable generalization.

## Conclusions

This study developed a country-scale machine learning workflow for LCOH screening across 54 harmonized African scenarios. The analysis used 14 standardized predictors capturing project scale (electrolyzer GW, renewables GW), infrastructure (storage Mm³, pipelines km), economics (investment $B), development stage (maturity ordinal 1–5), enabling context (energy security/sustainability scores 0–10), market orientation (export/domestic Mtpa), environmental impact (CO₂ reduction Mt/year), and resource constraints (water demand Mm³/year). The dataset construction followed a transparent 3-step harmonization protocol: priority reconciliation from national strategies > international assessments > project reports with ± 20% averaging, regional ratio imputation for missing values, and z-score standardization pre-training (denormalized for reporting). Hyperopt-optimized Gradient Boosting achieved top performance with R²=0.9762 on holdout validation (RMSE = 0.0840 EUR/kg) and R²=0.971 ± 0.032 on nested cross-validation. Bayesian-tuned XGBoost followed closely (R²=0.9713). Regularized linear models captured a substantial linear signal from scale and readiness indicators. Neural architectures underperformed due to the small tabular dataset (*n* = 54). Robustness against circularity was confirmed using base-only predictors (excluding derived H₂ production, CO₂ reduction, market/water demands): R²=0.942 holdout/0.782 ± 0.098 CV. LCOH ranged 3.75–5.60 EUR/kg (median 4.90), with clear maturity stratification: conceptual stages > 4.5 EUR/kg versus FID/construction < 4.2 EUR/kg.

SHAP explainability ranked renewable energy capacity, electrolyzer capacity, and energy security index as leading contributors to prediction variation. Higher values consistently predicted lower LCOH through scale economies and system readiness patterns, not causal mechanisms. Evidence-based SDG linkages emerged naturally: SDG7 via dominant renewable capacity importance (~ 0.40 SHAP contribution), SDG9 through energy security/infrastructure correlations, SDG13 indirectly via CO₂ tracking production scale, SDG6 critically through water demand scaling with investment/export potential, and SDG17 via model benchmarking stability enabling transparent decision support. This transferable screening pipeline supports data-scarce settings by prioritizing investment and linking cost patterns to SDG indicators. Predictions reflect scenario-based techno-economic assumptions, not empirical project outcomes. Future research requires longitudinal expansion, country/maturity holdout validation, uncertainty quantification through prediction intervals, and harmonized templates capturing electricity procurement structure, storage duration, grid constraints, and basin-level water stress to enhance transferability beyond continental Africa.

## Supplementary Information

Below is the link to the electronic supplementary material.

Supplementary Material 1

Supplementary Material 2

## Data Availability

The datasets generated and/or analyzed during the current study, along with the associated code, are available in the Hydrogen_AI repository: [https://github.com/tarekhemdan/Hydrogen_AI](https:/github.com/tarekhemdan/Hydrogen_AI).
