# Supplementary material for: Toward sustainable energy production: a comparative machine learning framework for predicting green hydrogen cost across the african continent
Source: Sci Rep. 2026 Apr 17;16:12855. doi: 10.1038/s41598-026-47726-w (PMC13096549; doi:10.1038/s41598-026-47726-w)
Supplement: Supplementary file 1 — Supplementary Material 1 [file 41598_2026_47726_MOESM1_ESM.docx]

Supplementary material of the article, titled “**Toward Sustainable Energy Production: A Comparative Machine Learning Framework for Predicting Green Hydrogen Cost Across the African Continent**”

**Table S1** Variable construction lineage for the synthetic dataset.

| **Variable name** | **Definition** | **Unit used in analysis** | **Targeted magnitude/ordering** | **Construction rule/transformation** |
| --- | --- | --- | --- | --- |
| Country | The name of an African country is included in the scenario | Text | See the countries in Table 1 | Fixed the list of country names. |
| LCOH_EUR_per_kg | Levelized cost of hydrogen at plant gate | EUR per kg | ≈3.8–5.6 EUR/kg, lowest in flagship/advanced countries | Assigned within tier-specific ranges, lower for flagship/advanced, higher for frontier.matthey+2 |
| Electrolyzer_Capacity_GW | Installed or planned electrolyzer capacity | GW | ≈0.08–15 GW, the highest in flagship countries | Drawn from tier-specific GW ranges, larger values for flagship/advanced tiers. |
| H2_Production_Capacity_Mtpa | Nominal annual green hydrogen production capacity | Mtpa | Proportional to electrolyzer capacity | Computed as electrolyzer capacity multiplied by assumed Mt H₂ per GW per year, following the conversion above. |
| Renewable_Energy_Capacity_GW | Renewable electricity capacity feeding hydrogen projects | GW | Higher than electrolyzer capacity, especially in flagship/advanced | Computed as a multiplier of electrolyzer capacity using tier-specific renewable multipliers. |
| CO2_Reduction_Mt_per_year | Annual CO₂ emissions avoided by hydrogen production | Mt per year | Proportional to hydrogen production, higher in large producers | Computed from H₂ production capacity using an assumed Mt CO₂ avoided per Mt H₂. |
| Storage_Capacity_Mm3 | Hydrogen storage volume | Mm³ | Increases with electrolyzer capacity and export orientation | Computed as electrolyzer capacity multiplied by a tier-specific storage-per-GW factor. |
| Distribution_Pipeline_km | Length of the hydrogen pipeline network | km | Longer in export-oriented and advanced countries | Sampled from tier-specific base ranges, with higher values for export-oriented tiers. |
| Energy_Security_Score | Composite index of energy system robustness and diversification | 0–10 score | Higher scores for diversified, RE-rich, less import-dependent systems | Assigned within tier-specific score ranges, reflecting qualitative energy system characteristics.sciencedirect+2 |
| Sustainability_Index | Composite index of environmental and socio-economic sustainability | 0–10 score | Higher scores in countries with strong RE, high CO₂ reduction, and more jobs | Assigned within tier-specific score ranges reflecting combined sustainability performance.revistamultidisciplinar+2 |
| Export_Potential_Mtpa | Maximum annual hydrogen volume available for export | Mtpa | Larger fractions of H₂ output in coastal/export-flagship countries | Computed as H₂ production capacity multiplied by a tier-specific export share. |
| Domestic_Demand_Mtpa | Expected domestic hydrogen consumption | Mtpa | Higher ratios in more industrialized and diversified economies | Computed as H₂ production capacity multiplied by a tier-specific domestic share. |
| Investment_Billion_USD | Cumulative investment associated with hydrogen assets | Billion USD | Increases with electrolyzer capacity, highest in flagship projects | Computed as electrolyzer capacity multiplied by a tier-specific investment-per-GW factor. |
| Project_Maturity_Stage | Qualitative stage of project development | Categorical text | “Concept” < “Feasibility” < “FEED/FID” < “FID/Construction” | Assigned by tier, with specific overrides (e.g., Egypt = “Multiple Stages”, Namibia = “FID/Construction”). |
| Water_Demand_Mm3_per_year | Annual water consumption for hydrogen production | Mm³ per year | Proportional to H₂ production; highest in large producers | Computed as H₂ production capacity multiplied by a tier-specific water-per-Mt H₂ factor. |

**The Python code:**

**# Python code used to construct the synthetic input data for Table 1**

# File name in SI: compile_dataset.py

#

# This script encodes the rules and parameter ranges that were used to

# generate the country-level variables, and write the resulting dataset

# to "SI_Table1_dataset.csv".

import pandas as pd

import numpy as np

# 1. Countries with region and qualitative development tier

countries = [

("Algeria", "North", "advanced"),

("Angola", "Central", "emerging"),

("Benin", "West", "emerging"),

("Botswana", "Southern", "emerging"),

("Burkina Faso", "West", "frontier"),

("Burundi", "East", "frontier"),

("Cameroon", "Central", "frontier"),

("Cape Verde", "West", "frontier"),

("Central African Republic", "Central", "frontier"),

("Chad", "Central", "frontier"),

("Comoros", "East", "frontier"),

("Congo", "Central", "frontier"),

("Democratic Republic Congo", "Central", "frontier"),

("Djibouti", "East", "emerging"),

("Egypt", "North", "advanced"),

("Eritrea", "East", "frontier"),

("Eswatini", "Southern", "frontier"),

("Ethiopia", "East", "emerging"),

("Gabon", "Central", "emerging"),

("Gambia", "West", "frontier"),

("Ghana", "West", "emerging"),

("Guinea", "West", "emerging"),

("Guinea Bissau", "West", "frontier"),

("Ivory Coast", "West", "emerging"),

("Kenya", "East", "emerging"),

("Lesotho", "Southern", "frontier"),

("Liberia", "West", "frontier"),

("Libya", "North", "emerging"),

("Madagascar", "East", "emerging"),

("Malawi", "Southern", "frontier"),

("Mali", "West", "frontier"),

("Mauritania", "West", "flagship"),

("Mauritius", "East", "emerging"),

("Morocco", "North", "advanced"),

("Mozambique", "Southern", "emerging"),

("Namibia", "Southern", "flagship"),

("Niger", "West", "frontier"),

("Nigeria", "West", "advanced"),

("Rwanda", "East", "frontier"),

("Sao Tome", "Central", "frontier"),

("Senegal", "West", "emerging"),

("Seychelles", "East", "frontier"),

("Sierra Leone", "West", "frontier"),

("Somalia", "East", "frontier"),

("South Africa", "Southern", "advanced"),

("South Sudan", "East", "frontier"),

("Sudan", "North", "frontier"),

("Tanzania", "East", "emerging"),

("Tanzania_alt", "East", "emerging"),

("Togo", "West", "frontier"),

("Tunisia", "North", "emerging"),

("Uganda", "East", "emerging"),

("Zambia", "Southern", "frontier"),

("Zimbabwe", "Southern", "frontier"),

]

# 2. Tier-specific parameter ranges encoding the scenario logic

tier_params = {

"flagship": {

"LCOH_range": (3.8, 4.2),

"electrolyzer_GW_range": (5.0, 15.0),

"RE_mult": (1.8, 2.2), # RE capacity ≈ multiplier of electrolyzer capacity

"CO2_per_MtH2": (16.0, 20.0), # Mt CO₂ avoided per Mt H₂

"storage_per_GW": (2.0, 3.0), # Mm³ per GW

"pipeline_base": (400.0, 900.0),# km

"ES_range": (8.0, 9.2), # energy security score

"SUST_range": (8.5, 9.2), # sustainability index

"export_share": (0.6, 0.8),

"domestic_share": (0.1, 0.2),

"inv_per_GW": (6.0, 8.0), # billion USD per GW

"water_per_MtH2": (60.0, 75.0), # Mm³ per Mt H₂

"stage": "FEED/FID",

},

"advanced": {

"LCOH_range": (4.0, 4.6),

"electrolyzer_GW_range": (1.0, 6.0),

"RE_mult": (1.3, 1.8),

"CO2_per_MtH2": (14.0, 18.0),

"storage_per_GW": (1.5, 2.5),

"pipeline_base": (300.0, 700.0),

"ES_range": (7.0, 8.5),

"SUST_range": (8.0, 8.8),

"export_share": (0.3, 0.6),

"domestic_share": (0.2, 0.3),

"inv_per_GW": (5.0, 7.0),

"water_per_MtH2": (55.0, 70.0),

"stage": "Feasibility",

},

"emerging": {

"LCOH_range": (4.4, 5.1),

"electrolyzer_GW_range": (0.3, 2.5),

"RE_mult": (1.2, 1.6),

"CO2_per_MtH2": (12.0, 16.0),

"storage_per_GW": (1.0, 2.0),

"pipeline_base": (150.0, 400.0),

"ES_range": (6.2, 7.5),

"SUST_range": (7.5, 8.5),

"export_share": (0.1, 0.4),

"domestic_share": (0.2, 0.4),

"inv_per_GW": (4.0, 6.0),

"water_per_MtH2": (50.0, 65.0),

"stage": "Feasibility",

},

"frontier": {

"LCOH_range": (4.8, 5.6),

"electrolyzer_GW_range": (0.08, 0.4),

"RE_mult": (1.0, 1.3),

"CO2_per_MtH2": (10.0, 14.0),

"storage_per_GW": (0.8, 1.5),

"pipeline_base": (50.0, 200.0),

"ES_range": (5.0, 6.2),

"SUST_range": (6.5, 7.5),

"export_share": (0.0, 0.1),

"domestic_share": (0.1, 0.3),

"inv_per_GW": (3.0, 4.5),

"water_per_MtH2": (45.0, 60.0),

"stage": "Concept",

},

}

# 3. Deterministic generation of variables using the above rules

rng = np.random.default_rng(42) # fixed seed for reproducibility

rows = []

for country, region, tier in countries:

p = tier_params[tier]

# LCOH (EUR/kg)

LCOH = float(rng.uniform(*p["LCOH_range"]))

# Electrolyzer capacity (GW)

elec_GW = float(rng.uniform(*p["electrolyzer_GW_range"]))

# H2 production capacity (Mtpa) from electrolyzer capacity

# Assume ~0.15–0.20 Mt H2 / GW / year

base_Mt_per_GW = rng.uniform(0.15, 0.20)

H2_Mtpa = elec_GW * base_Mt_per_GW

# Renewable energy capacity (GW) as multiple of electrolyzer capacity

RE_mult = rng.uniform(*p["RE_mult"])

RE_GW = elec_GW * RE_mult

# CO2 reduction (Mt/year) from H2 output and abatement factor

CO2_per_MtH2 = rng.uniform(*p["CO2_per_MtH2"])

CO2_Mt = H2_Mtpa * CO2_per_MtH2

# Storage capacity (Mm3)

storage_per_GW = rng.uniform(*p["storage_per_GW"])

storage_Mm3 = elec_GW * storage_per_GW

# Pipeline length (km)

pipeline_km = rng.uniform(*p["pipeline_base"])

# Energy security score and sustainability index (0–10)

ES = rng.uniform(*p["ES_range"])

SUST = rng.uniform(*p["SUST_range"])

# Export and domestic shares of H2

export_share = rng.uniform(*p["export_share"])

domestic_share = rng.uniform(*p["domestic_share"])

export_Mt = H2_Mtpa * export_share

domestic_Mt = H2_Mtpa * domestic_share

# Investment (billion USD)

inv_per_GW = rng.uniform(*p["inv_per_GW"])

investment_BUSD = elec_GW * inv_per_GW

# Water demand (Mm3/year) from H2 output

water_per_MtH2 = rng.uniform(*p["water_per_MtH2"])

water_Mm3 = H2_Mtpa * water_per_MtH2

# Project maturity stage (tier default with specific overrides)

stage = p["stage"]

if country == "Egypt":

stage = "Multiple Stages"

if country == "South Africa":

stage = "Feasibility"

if country == "Namibia":

stage = "FID/Construction"

rows.append([

country,

round(LCOH, 2),

round(elec_GW, 2),

round(H2_Mtpa, 3),

round(RE_GW, 1),

round(CO2_Mt, 1),

round(storage_Mm3, 1),

round(pipeline_km, 1),

round(ES, 1),

round(SUST, 1),

round(export_Mt, 3),

round(domestic_Mt, 3),

round(investment_BUSD, 1),

stage,

round(water_Mm3, 1),

])

# 4. Build DataFrame and write the Table 1 input file

columns = [

"Country",

"LCOH_EUR_per_kg",

"Electrolyzer_Capacity_GW",

"H2_Production_Capacity_Mtpa",

"Renewable_Energy_Capacity_GW",

"CO2_Reduction_Mt_per_year",

"Storage_Capacity_Mm3",

"Distribution_Pipeline_km",

"Energy_Security_Score",

"Sustainability_Index",

"Export_Potential_Mtpa",

"Domestic_Demand_Mtpa",

"Investment_Billion_USD",

"Project_Maturity_Stage",

"Water_Demand_Mm3_per_year",

]

df = pd.DataFrame(rows, columns=columns)

df.to_csv("SI_Table1_dataset.csv", index=False)

print("SI_Table1_dataset.csv written successfully.")

**
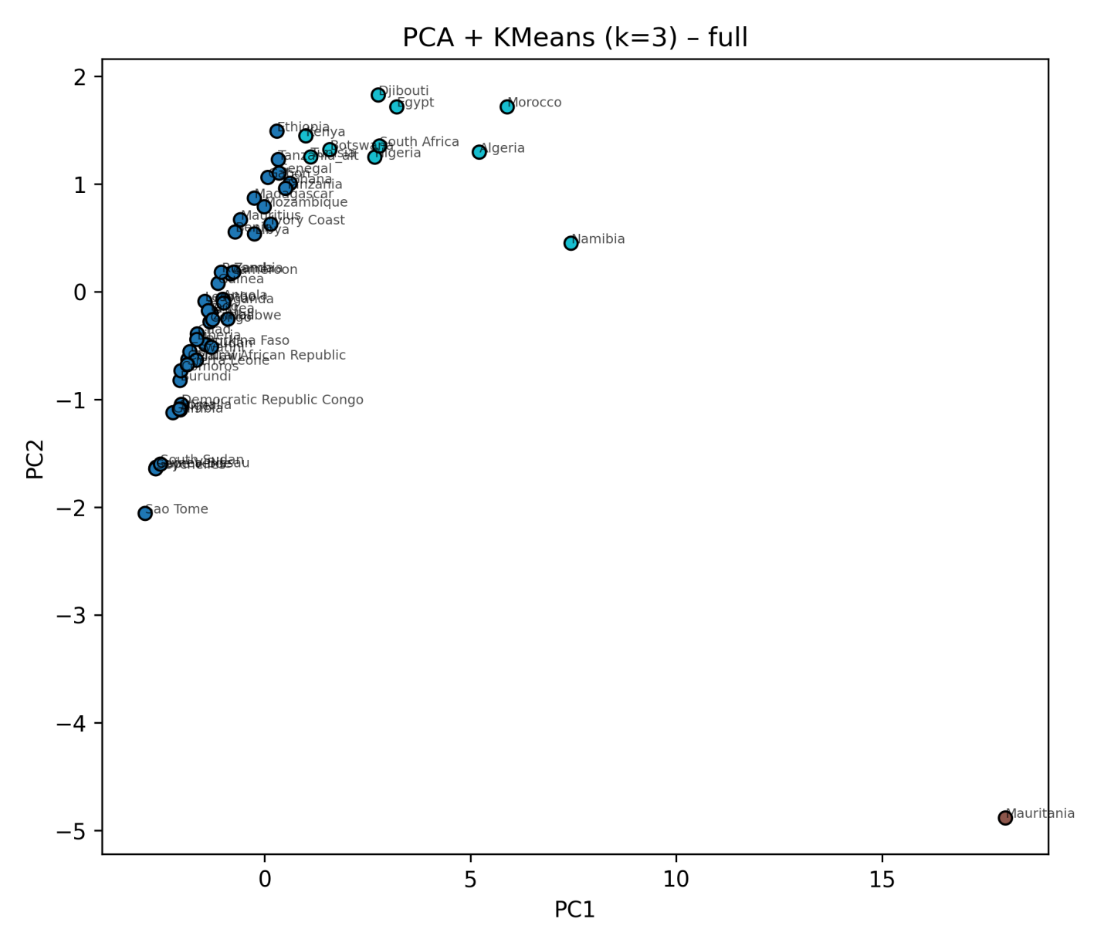
**

**Fig. S1** PCA (PC1–PC2) with K-means (k = 3) using the full predictor set (including derived variables).

**
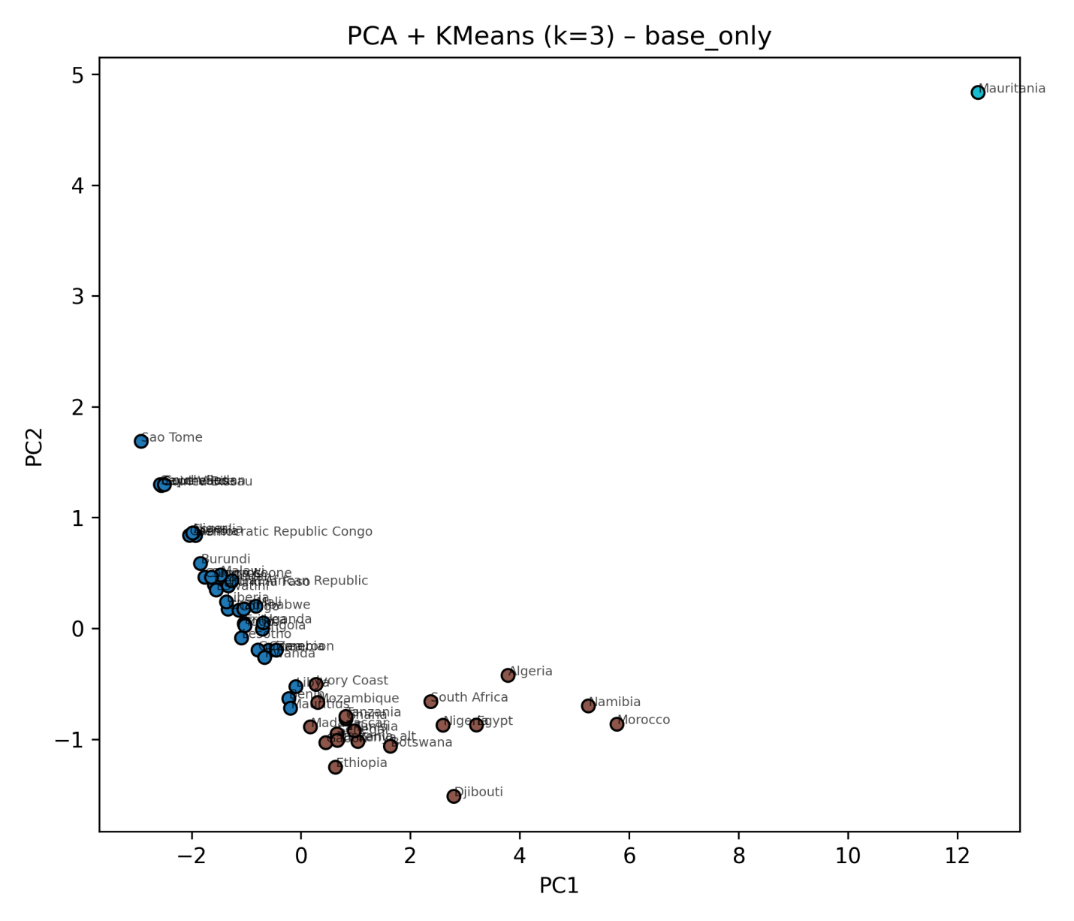
**

**Fig. S2** PCA (PC1–PC2) with K-means (k = 3) using base predictors only (derived variables excluded) for robustness.


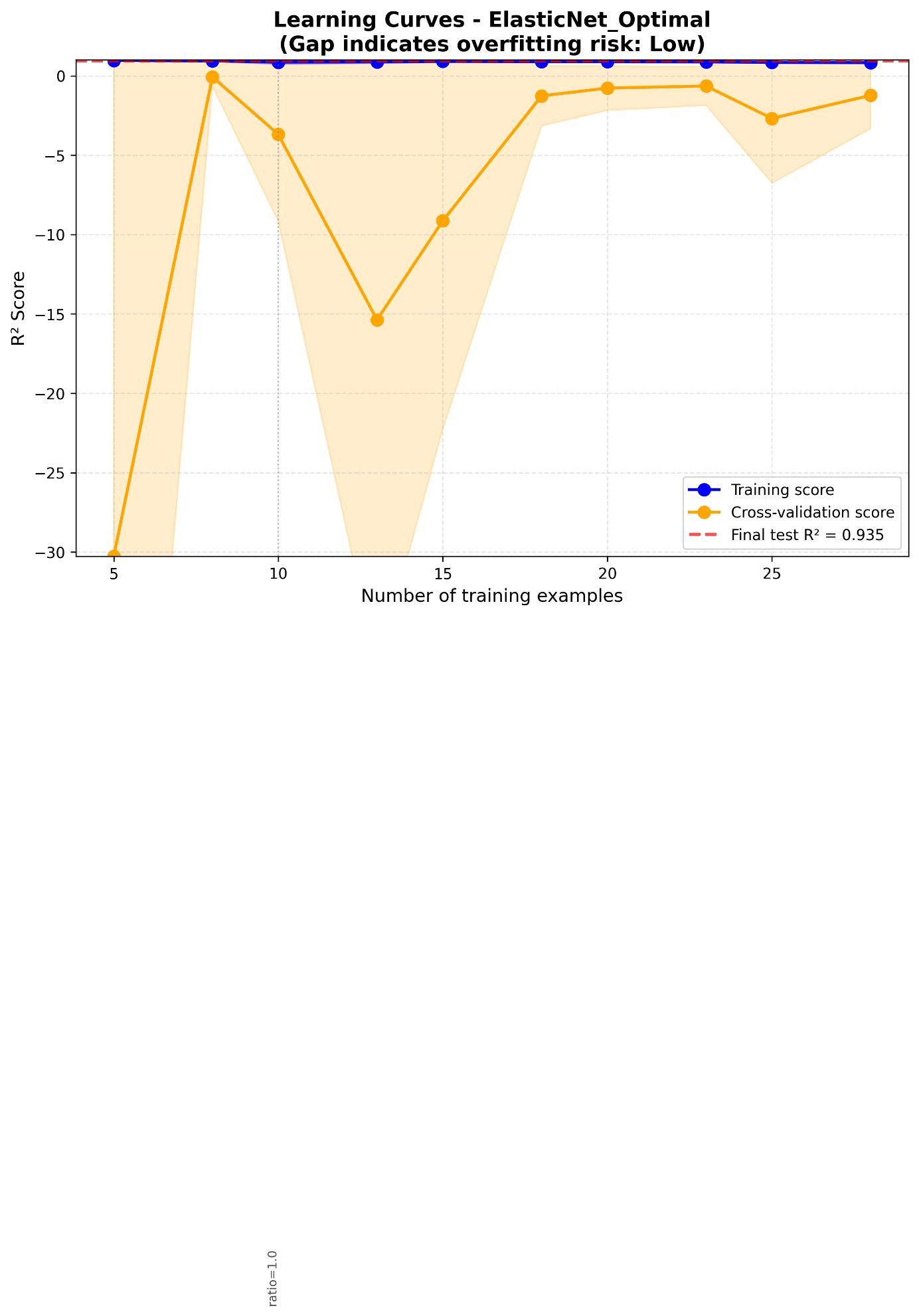


**Fig. S3** Learning curve for ElasticNet_Optimal (training and cross-validation R² vs. training size).


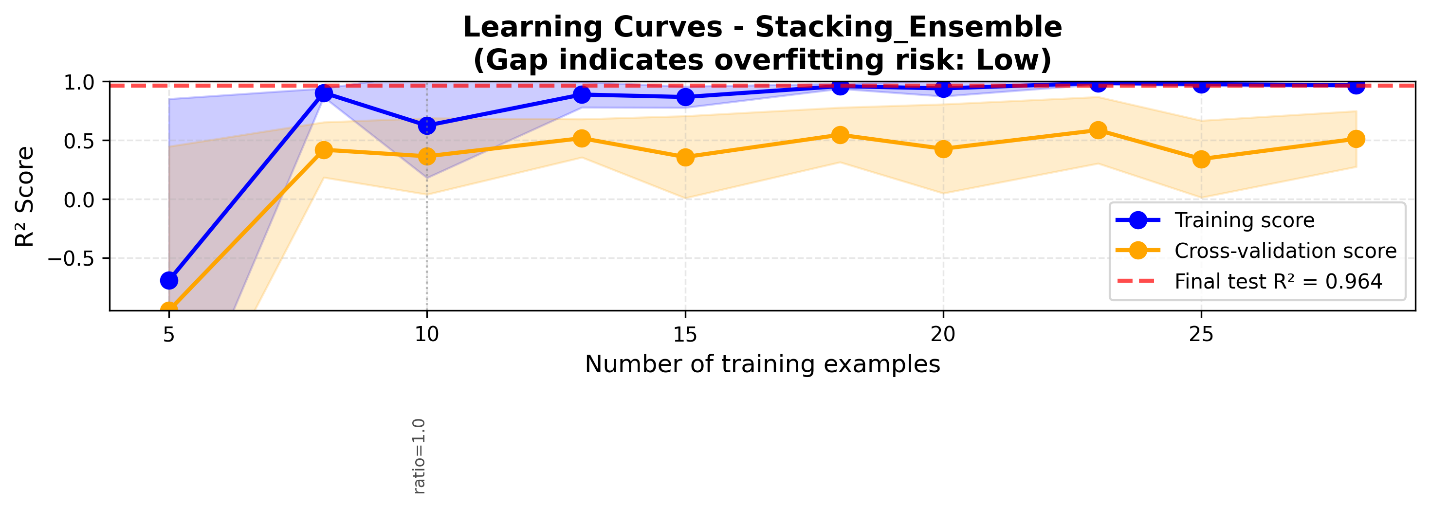


**Fig. S4** Learning curve for Stacking_Ensemble (training and cross-validation R² vs. training size).
