# Supplementary figures and images for: Toward sustainable energy production: a comparative machine learning framework for predicting green hydrogen cost across the african continent
Source: Sci Rep. 2026 Apr 17;16:12855. doi: 10.1038/s41598-026-47726-w (PMC13096549; doi:10.1038/s41598-026-47726-w)

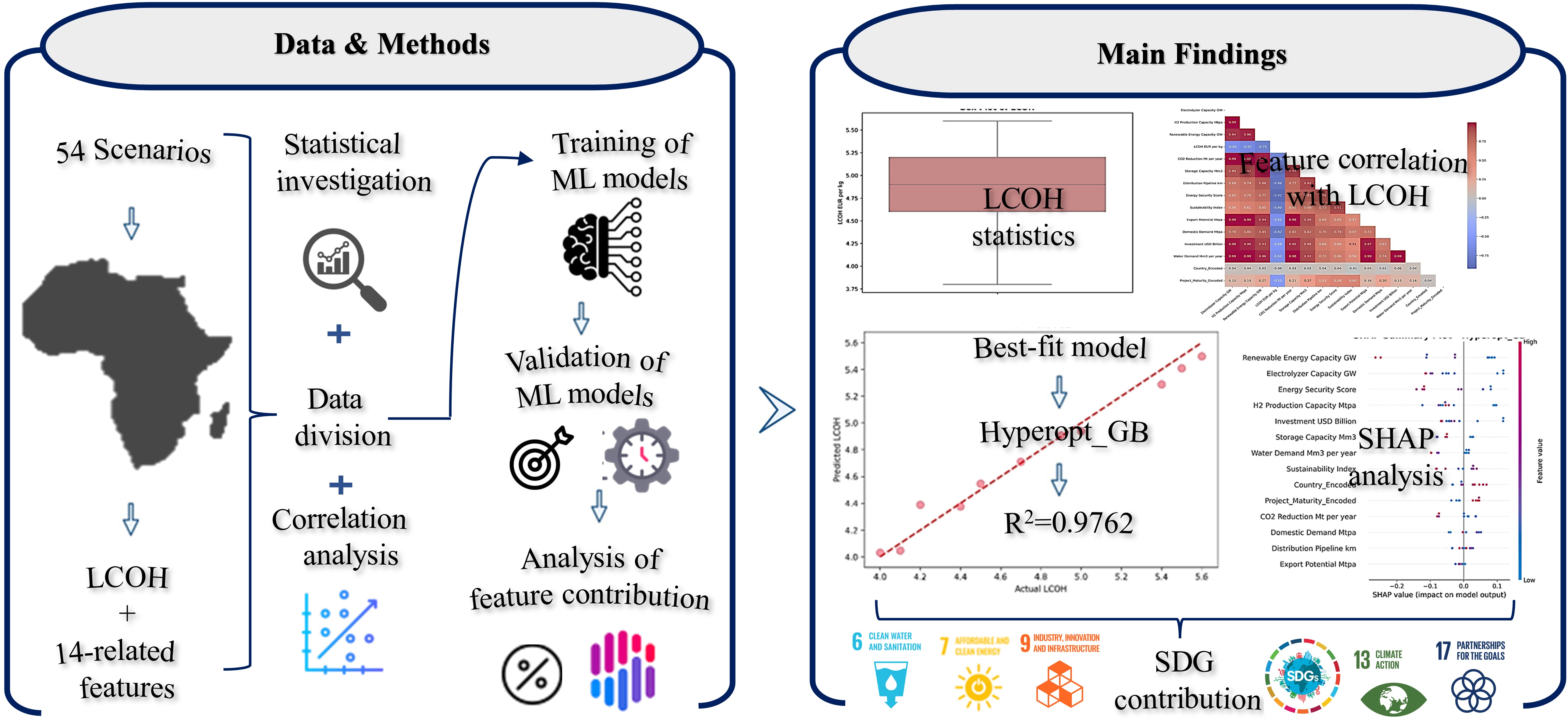

Supplement: Supplementary file 2 — Supplementary Material 2 [file 41598_2026_47726_MOESM2_ESM.jpg]
